# The carbon source-dependent pattern of antimicrobial activity and gene expression in *Pseudomonas donghuensis* P482

**DOI:** 10.1038/s41598-021-90488-w

**Published:** 2021-05-26

**Authors:** Marta Matuszewska, Tomasz Maciąg, Magdalena Rajewska, Aldona Wierzbicka, Sylwia Jafra

**Affiliations:** Laboratory of Plant Microbiology, Intercollegiate Faculty of Biotechnology of University of Gdansk and Medical University of Gdansk, University of Gdansk, Gdansk, Poland

**Keywords:** Bacterial genetics, Bacterial transcription, Reverse transcription polymerase chain reaction

## Abstract

*Pseudomonas donghuensis* P482 is a tomato rhizosphere isolate with the ability to inhibit growth of bacterial and fungal plant pathogens. Herein, we analysed the impact of the carbon source on the antibacterial activity of P482 and expression of the selected genes of three genomic regions in the P482 genome. These regions are involved in the synthesis of pyoverdine, 7-hydroxytropolone (7-HT) and an unknown compound (“cluster 17”) and are responsible for the antimicrobial activity of P482. We showed that the P482 mutants, defective in these regions, show variations and contrasting patterns of growth inhibition of the target pathogen under given nutritional conditions (with glucose or glycerol as a carbon source). We also selected and validated the reference genes for gene expression studies in *P. donghuensis* P482. Amongst ten candidate genes, we found *gyrB*, *rpoD* and *mrdA* the most stably expressed. Using selected reference genes in RT-qPCR, we assessed the expression of the genes of interest under minimal medium conditions with glucose or glycerol as carbon sources. Glycerol was shown to negatively affect the expression of genes necessary for 7-HT synthesis. The significance of this finding in the light of the role of nutrient (carbon) availability in biological plant protection is discussed.

## Introduction

Organic carbon is an essential source of energy for all heterotrophic (micro)organisms. To obtain energy, microorganisms can use, through various catabolic pathways, numerous organic compounds, ranging from simple carbohydrates or organic acids to complex polymers^1,2^. The available carbon source and genetic determinants of the microorganisms define the metabolic pathways employed in a given environment. This complex metabolic machinery of a cell complicates even more with each metabolic step since the metabolites resulting from the assimilation of a carbon source (as well as the carbon source itself) are further processed and may participate in regulation of gene expression and secondary metabolism of the cell^3–5^. Thus, environmental conditions such as abundance and availability of the carbon source may affect a myriad of essential bacterial cell processes, such as synthesis of antimicrobials^6,7^, or biofilm formation^8^.

*Pseudomonas donghuensis* P482, a tomato rhizosphere isolate, has been previously studied for its antimicrobial potential against bacterial^9,10^ and fungal^11^ plant pathogens. Like many other fluorescent pseudomonads^12^, P482 produces a pyoverdine siderophore and hydrogen cyanide, however, it lacks the genes essential for the synthesis of the compounds such as^10^: phenazines, 2,4-DAPG, pyrrolnitrin, pyoluteorin or (cyclic)-lipopeptides, necessary for the antimicrobial activity of other *Pseudomonas*^13^. P482 is one of only four strains of *P. donghuensis* species described so far^10,14–16^. Therefore, knowledge concerning its biology, genetics and ecology is still limited. Being a member of this recently established species^14,17^, little is known about the molecular mechanisms underlying the regulation of P482 gene expression and metabolism.

Recent studies of Chen *﻿et al.*^18^ and Muzio *﻿et al.*^19^ unveil the gene cluster of *P. donghuensis* HYS^T^ and SVBP6, respectively, responsible for the production of 7-hydroxytropolone (7-HT), which was shown to act both as a nonfluorescent iron chelator^20,21^ and an antifungal agent^19^*.* Our previous study identified a cluster of P482 genes (loci: BV82_4705-BV82_4712) being partially responsible for its antibacterial activity^10^. In silico analysis of this gene cluster proved high similarity to the 7-HT biosynthesis cluster of the HYS^T^ strain^18^. Thus, it allowed us to establish that 7-HT acts not only as an iron chelator and an antifungal compound but also is involved in antibacterial activity. Pyoverdines, fluorescent siderophores of many soil- and plant-associated microorganisms, including *Pseudomonas* spp., were reported to exhibit an antifungal activity due to their high iron affinity^22^. P482 produces a pyoverdine siderophore, which was not relevant for P482 antibacterial activity in lysogeny broth (LB)^10^. The type of carbon source and its availability was shown to affect the production of bacterial antimicrobials^7,23^ or siderophores^24,25^ in various bacterial genera, including *Pseudomonas*^6,26–29^. 7-HT has been described as the *P. donghuensis* iron scavenger relatively recently^20^, however, the antimicrobial activities of hydroxytropolones have been known earlier^30,31^. It has already been established that the genes of the 7-HT biosynthesis cluster in *P. donghuensis* are controlled by two regulatory systems: Gac-Rsm and LysR/TetR^18^, however, the information on the nutritional regulation of these genes is lacking.

In our previous work on P482, we identified another gene cluster, “cluster 17”, potentially involved in the synthesis of antimicrobial secondary metabolites^10^. Up to date, no information has been published concerning gene clusters of high similarity to the P482 “cluster 17”, however, our preliminary in silico research suggested a vague link with polyketide synthesis. Polyketides comprise a large group of highly biologically active metabolites, with many of them being used as antibiotics, antifungal agents or other commonly known drugs^32^. The provisional classification of the “cluster 17” genes possibly involved in the antibacterial activity of P482 prompted us to include them as the genes of interest in this study.

The differences in biosynthesis levels of secondary metabolites such as antimicrobial compounds under diverse environmental conditions are usually a direct result of the regulation of gene expression. Carbon source is one of the factors that can significantly influence gene expression and the importance of this effect on the secondary metabolism of bacteria resulted in numerous investigations on the subject^5,33,34^.

A golden standard method for gene expression studies is Reverse Transcription Quantitative PCR (RT-qPCR). Minimum Information for Publication of Quantitative Real-Time PCR Experiments (MIQE) guidelines^35^ were developed to help researchers generate exemplary gene expression data and reduce any bias in RT-qPCR studies. These guidelines emphasise the importance of a meticulous selection of reference genes (RGs) for the normalisation of gene expression data. Nevertheless, up to date, no literature data is available concerning the selection of RT-qPCR RGs in *P. donghuensis* species.

The presented study focuses on the differences in *P. donghuensis* P482 strain antimicrobial activity when cultured with the availability of a single type of carbon source (namely glucose or glycerol). We provide data showing that under specific conditions of carbon availability, a little-known gene cluster together with the pyoverdine biosynthetic genes are highly engaged in the antibacterial activity of P482, whereas the effect of the 7-HT biosynthetic pathway is negligible. To confirm our hypothesis on this carbon source-dependent shift in secondary metabolism, we performed RT-qPCR tests according to MIQE guidelines, preceded by selecting the most stably expressed P482 housekeeping genes to normalise further data. To the best of our knowledge, we present the first comprehensive research on the reference gene stability in *P. donghuensis* species and an RT-qPCR analysis showing that the choice of carbon source in the medium affects the expression of genes responsible for 7-HT biosynthesis by *P. donghuensis* P482.

## Results

### Carbon source dependency of siderophore-based P482 antibacterial activity

**Figure 1:**
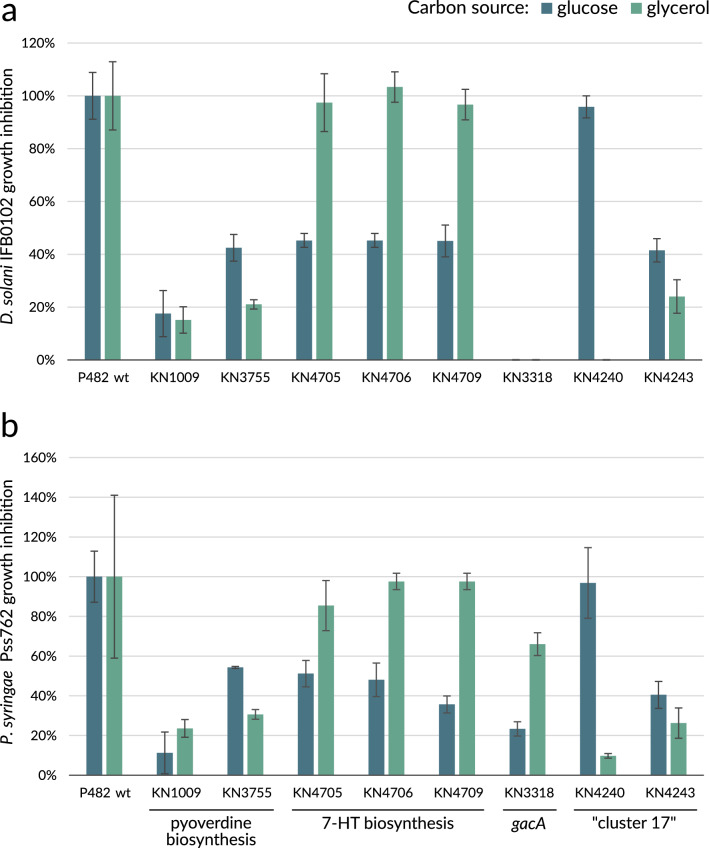
Growth inhibition of *Dickeya solani* IFB0102 (**a**) and *Pseudomonas syringae* pv. *syringae* Pss762 (**b**) by *Pseudomonas donghuensis* P482 mutants tested on minimal M9-agar medium with 0.4% glucose or 0.4% glycerol as a sole carbon source. The bars represent the percentage of the growth inhibition zone obtained for P482 wt under given conditions. *Pseudomonas vranovensis* DSM16006^T^ does not cause growth inhibition of the tested pathogens and was used as a negative control strain (see Supplementary Data Figure S1). The assay was performed in triplicates; error bars represent standard deviation.

The *P. donghuensis* P482 mutants with a previously confirmed deficiency in antibacterial activity^10^ (namely: KN3318, KN4705, KN4706, KN4709, KN1009 and KN3755) were analysed in a direct antibiosis assay against two plant pathogens: *Dickeya solani* IFB0102 and *Pseudomonas syringae* pv. *syringae* Pss762. The analyses were performed on M9-agar minimal media containing glucose or glycerol as a sole carbon source. Direct antibacterial activity of wild type (wt) P482 and its mutants was reflected in pathogen growth inhibition zones (Supplementary Data Figure S1). Their diameters were measured, and the results obtained for the mutants were normalised to the activity of the P482 wt and presented as a percentage of this activity (Fig. 1 and Supplementary Data Table S1).

The analysis revealed that all tested mutants but KN3318, a *gacA*^*−*^ mutant, show similar patterns of antibacterial capabilities regardless of the pathogen (Fig. 1). The KN1009 and KN3755 mutants, affected in pyoverdine synthesis, show highly suppressed antibacterial activity independently of carbon source or pathogen (Fig. 1a,b). This suggests an essential role of this siderophore in P482 antagonism towards bacterial plant pathogens under the tested conditions.

When glucose was the single carbon source in the growth medium, the antibacterial activity of the mutants with inactivated genes required for 7-HT biosynthesis (KN4705, KN4706, KN4709) was reduced to about 50% of the P482 wt activity. This is consistent with the fact that 7-HT is reported as one of the compounds responsible for antimicrobial activity in all *P. donghuensis* strains described so far^10,16,19^. However, when glycerol was the sole carbon source, the activity of these mutants was not impaired in comparison to the P482 wt activity. This indicates that under such conditions the 7-HT biosynthesis pathway is not involved in P482 antibacterial activity, but other factor(s) are taking over its role, as this activity is still evident even despite the inactivation of given 7-HT biosynthesis genes.

In the case of KN3318, a mutant in the *gacA* gene involved in the regulation of the 7-HT biosynthesis^18^, but not modulating pyoverdine biosynthesis^19^, its ability to inhibit *D. solani* growth is abolished upon both tested carbon sources (Fig. 1a). However, KN3318 activity constitutes about 25% and 65% of the P482 wt activity against *P. syringae* upon glucose or glycerol as the only carbon source, respectively (Fig. 1b)*.* The differences in the activity of KN3318 mutant might be explained by other factors produced by P482, e.g. in response to the pathogen’s cues, playing a role in growth inhibition of *P. syringae,* but not that of *D. solani.*

Taken together, these results suggest that the presence of alternative carbon sources, glucose or glycerol, in a minimal growth medium has an impact on *P. donghuensis* P482 antibacterial activity. The carbon sources tested affect the P482 antimicrobial activity dependent on the 7-HT biosynthesis but do not substantially influence its pyoverdine biosynthesis-dependent activity.

### Identification of a novel P482 genomic region potentially involved in the biosynthesis of an antimicrobial compound

In our former study on the antibacterial activity of *P. donghuensis* P482, we used the antiSMASH 2.0^36^ tool to identify potential regions in the P482 genome that could be involved in the synthesis of antimicrobials^10^. An initial in silico analysis of the clusters selected by the antiSMASH 2.0 revealed “cluster 17” being the only one that contained annotated open reading frames (ORFs) potentially involved in polyketide biosynthesis. Although this result was only obtained in the early version of antiSMASH (2.0) and was not repeated with its more recent versions (including 5.0), it prompted us to take an in-depth look at this gene cluster. The thorough analysis of this cluster unveiled that it consists of ten open reading frames (ORFs) (loci BV82_4236–BV82_4245) which are organised, according to the Operon-mapper platform^37^, into two operons (Supplementary Data Figure S2). The first operon comprises seven genes, the loci: BV82_4236, BV82_4237, BV82_4238, BV82_4239, BV82_4240, BV82_4241 and BV82_4242, and the second one only three loci: BV82_4243, BV82_4244 and BV82_4245. The GenBank IDs, annotations and gene orthology analyses for this genomic region are presented in Table 1.

**Table 1:** *P. donghuensis* P482 “cluster 17” annotation and features.

Locus (GenBank location)	Gene length (bp)	Product size (aa)	Annotation(s)^a^	KEGG [KO, EC]^b^	KEGG pathway^c^	Number of (GenBank) high score hits^d^
BV82_4236 (JHTS01000048.1: 16103–117158)	1056	351	NAD-dependent epimerase/dehydratase family protein (RfbD domain containing)	–	–	8
BV82_4237 (JHTS01000048.1: 117159–118430)	1272	423	*patA* putrescine aminotransferase	KO: K09251 (EC: 2.6.1.82)	ko00310 ko00330 ko01100 ko01120	> 100
BV82_4238 (JHTS01000048.1: 118427–118870)	444	148	Polyketide cyclase/dehydrase and lipid transport family protein (SRPBCC ligand-binding domain containing)	–	–	3
BV82_4239 (JHTS01000048.1: 118876–120021)	1146	382	Putative isobutylamine N-hydroxylase (CaiA domain containing [acyl-CoA dehydrogenase])	–	–	4
BV82_4240 (JHTS01000048.1: 120018–120845)	828	276	SDR family NAD(P)-dependent oxidoreductase	–	–	44
BV82_4241 (JHTS01000048.1: 120856–121848)	993	309	Fatty acid desaturase family protein	–	–	2
BV82_4242 (JHTS01000048.1: 121881–122684)	804	272	Hypothetical protein DUF3050 domain containing	–	–	4
BV82_4243 (JHTS01000048.1: 122765–123829)	1065	355	*emrA* efflux transporter RND family, MFP subunit (HlyD_D23 domain containing)	K03543	–	10
BV82_4244 (JHTS01000048.1: 123826–125400)	1575	525	*emrB* H + antiporter-2 family protein	K03446	–	61
BV82_4245 (JHTS01000048.1: 125364–126713)	1350	450	*tolC* Outer membrane TolC family protein	K12340	ko01501 ko01503 ko02020 ko03070 ko04626	6

^a^Combined data obtained using: IGS annotations, KEGG BlastKoala, NCBI CDD and InterPro annotations.

^b^KO: Kegg Orthologs, EC: enzyme classification; obtained using KEGG BlastKOALA tool.

^c^Pathways: ko00310—lysine degradation, ko00330—arginine and proline metabolism, ko01100—metabolic pathways, ko01120—microbial metabolism in diverse environments, ko01501—beta-lactam resistance, ko01503—cationic antimicrobial peptide (CAMP) resistance, ko02020—two-component system, ko03070—bacterial secretion system, ko04626—plant-pathogen interactions.

^d^ ≥ 90% query coverage; 75% identity to proteins of taxon *Pseudomonas* spp.

One ORF belonging to this cluster is annotated as a hypothetical protein and, as well as several other ORFs from this cluster, does not appear to have any investigated orthologs. For these ORFs, both nucleotide and protein BLAST searches suggest only a small number of closely related genes (fewer than 10), indicating the nonconserved nature of this genomic region.

Based on the results obtained via in silico analysis, we performed site-directed mutagenesis to inactivate two genes in this cluster: locus BV82_4240 (with predicted product SDR family NAD(P)-dependent oxidoreductase) and locus BV82_4243 [with predicted product efflux transporter, RND family, MFP subunit (*emrA/hlyD*)] (Table 1). The two mutants obtained, namely KN4240 and KN4243, represent the genes of each operon of the “cluster 17”. In a preliminary study, the KN4240 and KN4243 mutants were tested for their antimicrobial activity upon an undefined LB-agar medium (Supplementary Data Figure S3). Their activity against *Dickeya solani* IFB0102 and *Pseudomonas syringae* pv. *syringae* Pss762 was relatively similar to that of P482 wt activity, although there was a clear tendency of lower activity of KN4243.

The KN4240 mutant revealed contrasting outcomes regarding the influence of carbon source (glucose or glycerol) on its antibacterial capability towards both pathogens when tested on M9 minimal medium (Fig. 1). When glucose was the sole carbon source for the KN4240 mutant, no differences in its antibacterial activity towards the *D. solani* IFB0102 strain were observed with respect to P482 wt. However, glycerol as a carbon source caused a total loss of antibacterial activity towards this pathogen (Fig. 1a). In the case of *P. syringae* pv. *syringae* Pss762 (Fig. 1b), we observed about 90% loss of activity of KN4240 mutant when glucose was changed to glycerol. These results imply that the product of the gene from locus BV82_4240 of the first operon in the “cluster 17” participates in the P482 wt antagonism towards both pathogens and is a key factor influencing the P482 antimicrobial activity when glycerol is the only available carbon source in the environment.

The antibacterial activity of the KN4243 mutant showed about 40% of the P482 wt strain activity against both pathogens when glucose was the sole carbon source and even lower (ca. 30%) activity when glycerol was used. This suggests that the efflux pump transport protein (encoded in locus BV82_4243) might facilitate the antibacterial activity of P482 wt.

These results suggest that carbon source largely affects the part of P482 antimicrobial activity determined by the genes of the “cluster 17”. The total loss of this activity in KN4240 in glycerol points to this operon as the main determinant of the antimicrobial activity of P482 under this condition. The gene in locus BV82_4243 is also associated with P482 antagonism towards plant pathogens, possibly by encoding an efflux pump transporting the antibacterial compound outside the cell, however, the antimicrobial activity of the KN4243 mutant is not particularly influenced by carbon sources investigated in this study.

### Selection of potential reference genes (RGs) for RT-qPCR

The potential reference targets for *P. donghuensis* P482 gene expression were chosen based on literature data^38–41^ to represent housekeeping genes coding for proteins from different functional groups*.* Ten candidate genes, namely: *acpP*, *algD*, *gyrB*, *lexA*, *mrdA*, *proC*, *recA*, *rpoB*, *rpoD* and *tuf* were selected for the analysis of their expression stability (loci and annotation of the candidate genes can be found in Supplementary Data Table S2). All tested genes are typically used as RGs in *Pseudomonas* aside from *tuf,* which was explored due to its selection as an RG in reports concerning Gram-positive bacteria^42,43^, whereas we found no data regarding its expression stability in *Pseudomonas* or other Gram-negative bacteria.

The primers were designed to amplify 120–153 bp fragments of the candidate reference genes (Supplementary Data Table S2). Their specificity was assessed with PCR product gel electrophoresis and melting curves for each reaction. Efficiency values of the designed primer pairs were between 96.4%-111.2%.

### Expression stability of the candidate RGs

**Figure 2:**
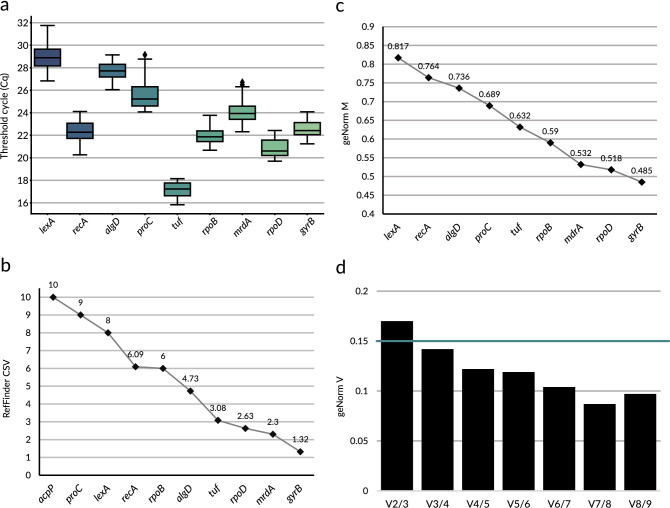
RT-qPCR reference gene selection for *Pseudomonas donghuensis* P482. (**a**) Boxplot representing the distribution of Cq (threshold cycle) data among the tested potential reference genes. The plot was calculated from raw data consisting of technical replicates’ mean Cq for each sample. The band in the box represents the median value, the top edge of the box is the upper quartile (Q3) while the bottom edge of the box is the lower quartile (Q1). Q3 and Q1 referred to the 75th percentile and the 25th percentile, respectively, meaning that 75 or 25% of the data were at or below the point. The whiskers represent the maximum and minimal values excluding outliers. Outlier data is presented with black diamond symbol (♦). (**b**) RefFinder comprehensive stability value (CSV) calculated as a geometric mean of the ranks assigned to the tested RGs by algorithms comprising RefFinder tool. The lower the CSV, the more stable the expression of a given gene. (**c**) qbase + geNorm RG stability analysis. Average expression stability of tested RGs obtained with geNorm algorithm shown as geNorm M value. The lower the M value, the more stable the gene expression. (**d**) Determination of the optimal number of reference targets shown as qbase + geNorm V chart for the tested reference targets. Analysis shows no significant difference in experimental situation when comparing the use of 3 or 4 reference genes (geNorm V < 0.15 for V3/4), meaning 3 reference genes are sufficient for expression normalisation.

The expression stability of all selected RGs was established under 12 conditions differing in nutritional composition, bacterial culture growth phase and temperature (Table 2). P482 wt was cultured under each of the conditions in three biological replicates, with the exception for the medium with tomato root exudates when two replicates were performed. qPCR was carried out on cDNA obtained after reverse transcription of total RNA extracted from the cultures. The results of each run were recorded and visualised using CFX Maestro software (BioRad, USA) for preliminary quality control. The qPCR results obtained were processed with the qbase + software (Biogazelle, Ghent, Belgium) and RefFinder engine to calculate the expression stability of each RG. Raw C_q_ results were plotted in a box plot (Fig. 2a) to visualise the C_q_ data distribution for the potential RGs.

**Table 2:** Conditions of *P. donghuensis* P482 culture used in reference gene selection study.

No.	Medium	Culturing time, temperature, and growth phase
1.	M9 + 0.4% glucose	12 h, 28 °C, late log phase
2.	M9 + 0.4% glucose + 30 µM FeSO_4_	12 h, 28 °C, late log phase
3.	M9 + 0.4% glucose + 30 µM FeCl_3_	12 h, 28 °C, late log phase
4.	M9 + 0.4% glycerol	30 h, 28 °C, late log phase
5.	M9 + 0.4% glycerol + 30 µM FeSO_4_	22 h, 28 °C, late log phase
6.	M9 + 0.4% glycerol + 30 µM FeCl_3_	30 h, 28 °C, late log phase
7.	M9 + 0.4% glucose + maize root exudates	12 h, 28 °C, late log phase
8.	M9 + 0.4% glucose + tomato root exudates	12 h, 28 °C, late log phase
9.	10% TSB	10 h, 28 °C, mid-log phase
10.	10% TSB	15 h, 28 °C, stationary phase
11.	10% TSB	16 h, 22 °C, late log phase
12.	LB	16 h, 28 °C, late log phase

RefFinder, the online tool, calculated and ranked the RGs stability with the use of 4 algorithms, namely ΔCt, BestKeeper, NormFinder and geNorm. This tool did not evaluate the quality of the data obtained or normalise the data to an interrun standard, as only the raw threshold cycle (Cq) data was entered for each gene. Thus, it was only employed in this study as a preliminary and supplementary implement. Each of the algorithms evaluated the stability of the RGs’ expression differently, which yields different results (Supplementary Data Figure S4). Despite this fact, their stability ranks are relatively consistent, placing *gyrB*, *rpoD*, *tuf* and *mrdA* as the most stably expressed genes in P482. The ranks established with each of the 4 algorithms were used to determine the RefFinder comprehensive stability value (CSV) for every tested gene, calculated as a geometric mean of the four ranks (Fig. 2b and Supplementary Data Table S3). The RefFinder ranking found *gyrB* the most stable of all tested genes (CSV = 1.32), the second-ranking stability value was obtained for *mrdA* (CSV = 2.3) and the third one for *rpoD* (CSV = 2.63).

These results were confronted with the outcome of calculations performed in the qbase + program. This software utilises the geNorm algorithm, which was also included in RefFinder analysis, but it takes into consideration several factors that are missing from RefFinder’s geNorm. The input consisted not only of raw C_q_ data, but also quality control data (negative and positive controls C_q_), interrun calibrator data for each run, primer efficiency data (standard curve) and sample specification (which allowed for control of the replicates’ quality). Using such data, the qbase + software calculated geNorm M values representing the expression stability of each of the tested RGs (Fig. 2c). One of the potential RGs, namely *acpP*, was dismissed from the qbase + geNorm analysis due to insufficient data quality (replicate variability higher than 0.3 cycle). The obtained results were consistent with the previously mentioned RefFinder RGs ranking: *gyrB* was found to be the most stably expressed among the selected genes with the geNorm M value equal 0.485, the second and third-ranking genes: *rpoD* and *mrdA* with M values equal 0.518 and 0.532, respectively.

Moreover, geNorm algorithm implemented in the qbase + software also calculates geNorm V value (Fig. 2d), suggesting the number of RGs (minimum two) that should be included into the analysis to give the most optimal normalisation factor (NF). geNorm V value is calculated by comparing how much the final normalised results would change if another RG was included in the analysis. The interpretation of the geNorm V values obtained in this study suggests that the addition of the third gene to the calculation of NF (geNorm V2/3 = 0.17) changes the results significantly (geNorm V > 0.15), therefore it is important to include the third reference gene in the experiment, whereas adding the fourth one (geNorm V3/4 = 0.142) would not change the outcome of the analysis significantly (geNorm V < 0.15).

Following the general conclusion from the presented results of the RGs selection, all further experiments concerning *P. donghuensis* P482 gene expression were performed with normalisation to the three most stably expressed reference genes: *gyrB*, *rpoD* and *mrdA*.

### Changes in the expression level of the selected genes of P482 in response to glucose or glycerol as a sole carbon source

Considering the differences in the carbon source-dependent antimicrobial activity of the tested mutants (namely: KN1009, KN3755, KN4705, KN4706, KN4709, KN3318, KN4240 and KN4243), we have analysed the expression of the respective genes in response to the carbon source present in the growth medium.

The RT-qPCR was performed in order to determine the expression level of the genes involved in pyoverdine (loci: BV82_1009 and BV82_3755), and 7-HT synthesis (loci: BV82_4705, BV82_4706 and BV82_4709), the *gacA* gene encoding a response regulator of GacS/GacA two-component system (locus BV82_3318) and the selected genes of “cluster 17” (loci: BV82_4240 and BV82_4243) in the presence of glucose or glycerol as a sole carbon source. P482 wt culturing time was established prior to RNA isolation with the measurement of the growth rate (Supplementary Data Figure S5) to avoid the influence of the growth phase on the gene expression and maintaining carbon source as the only variable. Glucose, while not being the preferred carbon source for *Pseudomonas* spp.^44^, is used immediately as the only carbon source (which is also true in *P. donghuensis* P482). However, glycerol causes a lag phase, which under the conditions applied, lasts about 12–16 h (depending on the mutation). Hence, the culturing time was extended correspondingly when using the M9 medium with glycerol. We observed that P482 wt and the mutants cultured in glycerol with regard to biomass yield could be divided into two groups. The first group includes KN3318, KN1009, KN4240 and KN4706, the cultures of which have reached an OD_600_ c.a. 0.35 in the stationary phase, and the second one, containing P482 wt and mutants KN3755, KN4709, KN4243, KN4709, of a lower optical density of the culture reaching approx. 0.2.

Primers designed to amplify the genes of interest listed in Supplementary Data Table S2, were tested for their specificity by melting curve analysis (Supplementary Data Figure S6). Primer efficiency was assessed using standard curves and was in the range from 95 to 107% (Supplementary Data Table S4). The expression data points obtained for all the genes of interest were normalised in respect to the expression of three RGs: *gyrB, mrdA* and *rpoD* for every tested sample (Supplementary Data Table S5).

**Figure 3:**
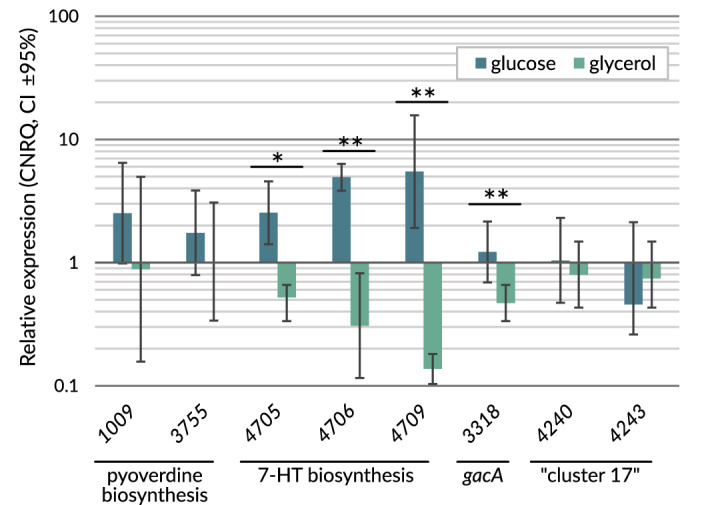
Comparison of relative expression (scaled to the mean CNRQ value calculated for each gene) of the chosen *P. donghuensis* P482 genes for bacteria cultured in the presence of glucose or glycerol as a sole carbon source in minimal medium M9. Error bars represent the 95% confidence interval (CI ± 95%). Statistically significant change in expression related to the particular carbon source used was observed for genes (the expression fold change value is given in brackets): 4709 (39.91), 4706 (16.01), 4705 (4.87), 3318 (2.6). Statistical analysis was performed using Student’s t-test, *) *p* < 0.05 **) *p* < 0.03. For clarity of the figure, loci references are represented by numbers only.

The expression level of the genes at loci BV82_1009 and BV82_3755 revealed no significant changes under both tested conditions (Fig. 3). This is in line with the results obtained for antibacterial activity of the corresponding mutants (KN1009 and KN3755), where only a slight difference in the antimicrobial activity was observed irrespective of the carbon source used (Fig. 1).

Interestingly, the results obtained for the selected genes involved in the 7-HT biosynthesis (loci BV82_4705, BV82_4706 and BV82_4709) demonstrated significant differences of the expression level depending on the carbon source used (Fig. 3). For BV82_4705 and BV82_4706, the expression was fivefold and 16-fold lower, respectively, on glycerol as a sole carbon source than on glucose (t-test results of expression comparison can be found in Table 3). The most significant difference was observed for the BV82_4709 gene, the expression of which was over 39 times lower on glycerol than on glucose. This is in line with the results obtained for the antibacterial activity of the KN4705, KN4706 and KN4709 mutants on glycerol which was comparable with that of P482 wt (Fig. 1).

**Table 3:** Student t-test results for the comparison of expression of P482 target genes under various carbon source (glucose or glycerol).

Target	*p*	Fold change glucose/glycerol	95% CI low	95% CI high
BV82_4709	0.02468	39.91	15.15	105.14
BV82_4706	0.02468	16.01	6.06	42.27
BV82_3318	0.02468	2.60	1.62	4.15
BV82_4705	0.02828	4.87	2.25	10.54
BV82_1009	0.18888	2.85	0.68	11.94
BV82_4243	0.26789	0.61	0.26	1.46
BV82_3755	0.26789	1.71	0.67	4.36
BV82_4240	0.35543	1.30	0.67	2.53

The expression of the *gacA* gene (locus BV82_3318) was significantly lower (2.6-fold difference) when P482 utilised glycerol instead of glucose as a carbon source. This result stands in agreement with antibiosis outcomes for KN3318 mutant, which remained active (more than 60% of the P482 wt activity) towards *P. syringae* upon glycerol. It suggests a reduced role of *gacA* in the overall antimicrobial activity of P482 upon glycerol as the only carbon source. This result is in agreement with the gene expression results obtained for the 7-HT biosynthesis genes, which are positively regulated by the Gac-Rsm system and their expression is also decreased on glycerol.

Furthermore, the expression of the genes in loci BV82_4240 and BV82_4243 did not change significantly due to the carbon source (Fig. 3). This result is somewhat surprising for BV82_4240, as upon glycerol, the corresponding mutant KN4240 demonstrated no or highly reduced antibacterial activity (Fig. 1).

The expression of the gene BV82_4243 did not change upon different carbon sources, and the corresponding mutant KN4243 demonstrated no significant difference in the antimicrobial performance, which is consistent with the RT-qPCR result.

Taken together, glycerol as a sole carbon source highly suppresses the expression of 7-HT biosynthesis genes in P482, which is confirmed by the direct antibiosis of the corresponding mutants. A slight attenuation of the gene expression by glycerol was also shown for the *gacA* gene, encoding the GacA regulator, which is a part of a system that positively modulates the 7-HT synthesis. However, even though we showed that the BV82_4240 gene from “cluster 17” plays a key role in the antibacterial activity of P482 upon glycerol, its expression was not affected by growth on alternative carbon sources. No significant fluctuation in gene expression in response to different carbon sources was observed for the genes responsible for pyoverdine biosynthesis (BV82_1009 and 3755) nor for BV82_4243, potentially important for transporting the antibacterial compound outside the cell.

## Discussion

### Siderophore biosynthesis as an important but not the only pathway of P482 antibacterial activity

Antibacterial activity of the environmental isolates of pseudomonads has been studied in terms of the biological control of plant pathogens for a few decades. Numbers of strains were reported to inhibit the growth of fungal^45–49^ and, to some extent, bacterial^9,50–53^ plant pathogens. One of the essential features of harmless pseudomonads (apart from the production of antimicrobials), which qualifies them as potential biological control agents, is their prevailing iron acquisition system^12^. *Pseudomonas* spp. are known to produce potent iron chelators, pyoverdines, giving their producers an advantage in iron-deficient environments^54^. Strains producing pyoverdines often succeed in competition with other microorganisms, as the iron scavengers contribute to the environmental fitness and antimicrobial activity of their producer^55^.

Herein, our exploration of the influence of a carbon source on the antibacterial activity of P482 and the expression of the selected genes involved in this activity is presented. Our data show that under minimal nutrient conditions, with limited iron availability, mutants affected in pyoverdine biosynthesis (KN1009, KN3755) exhibit a low level of antibacterial activity when compared to this of P482 wt (Fig. 1). This occurs regardless of the carbon source and might be attributed to pyoverdine providing an indirect antimicrobial effect, most likely as a consequence of the competitive mechanism of scavenging iron from the environment. These results are in contrast to those obtained for the same mutants under nutrient-rich conditions (LB-agar or Tryptic Soy agar, TSA)^10^, where no statistically significant decrease of antibacterial activity was observed, with respect to P482 wt. These observations remain in line with the fact that the lack of easily accessible iron in the environment stimulates the production of pyoverdine^56^. What is more, the study on the role of AlgRZ, the two-component regulatory system of *Pseudomonas aeruginosa* PAO1, on pyoverdine and pyocyanin production revealed that under iron-limiting conditions, changes in carbon utilisation influence the production of pyoverdine in this strain^57^. This finding supported the study of Sasnow *﻿et al.*^58^*,* where changes in carbon utilisation had an impact on pyoverdine production in the *Pseudomonas putida* KT2440 strain. Thus, carbon metabolism contributes to the regulation of the pyoverdine synthesis in P482 under limited iron availability and thereby plausibly indirectly influences antimicrobial activity.

Interestingly, in contrast to the pyoverdine mutants, the strains defective in the genes involved in 7-HT biosynthesis displayed a different pattern of antibacterial activity (Fig. 1). 7-HT belongs to tropolones, non-benzenoid aromatic compounds characterised by a seven-membered ring structure^59^. The tropolones, including hydroxytropolones, were found to be commonly produced by plants^60,61^ and fungi^62–64^, and in some cases by bacteria^18,65,66^. Tropolone from “*Pseudomonas plantarii*” was reported to cause disease symptoms on rice seedlings, such as chlorosis, root growth inhibition and wilting of the seedlings, which were also described for the pathogen itself^67^. However, the tropolones and their derivatives have been mainly described as possessing biological activities, *viz.* insecticidal, antimicrobial, antiviral and antitumor^68–70^. 7-HT is considered to be the primary antimicrobial of *P. donghuensis* strains, being responsible both for antibacterial and antifungal properties of all *P. donghuensis* strains described so far^10,16,19,20^. The antimicrobial activity of 7-HT has been attributed to two features of this low-molecular weight compound: (i) it is proven to be an inhibitor of enzymes, namely it suppresses the activity of the aminoglycoside-inactivating enzyme^71^ and inositol monophosphatase^72^; or (ii) it acts as an iron chelator which possibly gives the advantage to 7-HT producers in iron-deficient environments^20^. Our previous study showed that the 7-HT biosynthesis pathway contributes to the antibacterial effect of P482 against the selected strains of the *Dickeya* and *Pectobacterium* genera^10^ under highly nutritional conditions such as LB-agar or Tryptic Soy agar, since the 7-hydroxytropolone mutants (KN4705, KN4706 and KN4709) lost their antibacterial activity. Unexpectedly, the use of different carbon sources (glucose or glycerol) in the minimal medium had a substantial impact on the antibacterial activity of these mutants. The presence of glycerol, unlike glucose, almost completely restored the antibacterial activity of KN4705, KN4706 and KN4709 compared to P482 wt (Fig. 1). This suggests that under such conditions, another (alternative) pathway for the synthesis of antimicrobials is utilised, and as our further investigation shows, it might be linked to both pyoverdine and the product of “cluster 17” genes. It is noteworthy that the antimicrobial contribution of the genes of the three proposed pathways (inactivated in the mutants) does not appear to be additive. Therefore, we can speculate about some kind of cross-regulation or connection between these pathways, however, further research is needed to establish the nature of this relationship. Another interesting observation is the much more pronounced overall activity of P482 in the presence of glucose (reflected in the diameter of the growth inhibition zones 12.7–13.0 mm; Supplementary Data Table S1) than glycerol (5.3–5.8 mm). This poses a valid research question concerning the cause of this phenomenon, since it might be related to glycerol acting on P482 itself, but also affecting the P482-pathogen interaction.

### Novel gene cluster involved in the production of antimicrobial secondary metabolites

The results obtained in the course of this study indicate that a previously uninvestigated cluster of genes (here referred to as the “cluster 17”) is involved in *P. donghuensis* P482 antibacterial activity, particularly when P482 is cultured on glycerol as the sole carbon source. The gene in locus BV82_4240, a part of the “cluster 17”, is distinguished by its notably strong contribution to the antibacterial activity against the tested pathogens, as its inactivation resulted in the lack of or diminished antibacterial activity of the corresponding P482 mutant (KN4240) on glycerol (Fig. 1). The gene in locus BV82_4240 encodes a product belonging to a large superfamily of short chain dehydrogenases/reductases (SDR). SDRs are present in every living organism and catalyse various reactions belonging to both primary and secondary metabolism^73^. SDRs have been reported to take part in biosynthesis pathways of several antimicrobial compounds, such as polyketide antibiotic kalimantacin from *Pseudomonas fluorescens* BCCM_ID9359^74^ or fusidic acid in fungi from genus *Aspergillus*^75^ A gene encoding for an SDR can also be found in the 7-HT biosynthesis cluster in the P482 genome^10^. Hence, it could be hypothesised that the predicted P482 SDR encoded in the BV82_4240 locus is one of the enzymes involved in a pathway of biosynthesis of an antagonistic compound produced by this strain. Our finding, while preliminary, implies that this compound is likely distinct from 7-HT and pyoverdine (Supplementary Data Figure S7) and we suggest the possibility of a novel antimicrobial pathway should be taken into consideration in future studies on the antimicrobial activity of *P. donghuensis*.

The locus BV82_4243 is another gene of the “cluster 17” investigated in this study. It has been annotated as the conserved HlyD domain protein that might be a part of an efflux pump (EmrA-like protein). Together with the gene in locus BV82_4244 encoding a protein of high similarity to the EmrB from *Escherichia coli*^76^, and a third gene (BV82_4245), encoding a TolC-like protein, it constitutes the efflux pump operon. The bacterial TolC is a trimeric barrel protein structure that forms a channel in the outer membrane of a bacterial cell and is commonly found in Gram-negative bacteria^77^. Altogether, TolC and its corresponding inner membrane translocases, e.g., EmrAB, constitute the export system necessary to transport a diverse array of compounds with little chemical similarity and varied functions^78^. The EmrAB-TolC system belongs to the major facilitator superfamily (MFS) of efflux pumps and has been shown to provide bacterial resistance to antibiotics^79^. Although this finding was later questioned^80^, this type of pumps was also speculated to participate in *E. coli* siderophore enterobactin synthesis^76^. In *P. donghuensis,* the inactivation of the gene in locus BV82_4243 resulted in reduced antibacterial activity of the KN4243 mutant (on both carbon sources). Thus, it might be involved in the export of molecule(s) responsible for the antibacterial activity of P482. Moreover, spectrophotometric analyses show no presence of pyoverdine in the post-culture filtrates of KN4243 (no detectable peak at 405 nm) (Supplementary Data Figure S7). This indicates that the putative MFS efflux pump might be responsible for P482 pyoverdine secretion. Literature provides information on the secretion of pyoverdine in pseudomonads with the use of ABC-type pumps (PvdRT-OpmQ)^81,82^, RND-type pumps (MtdABC-OpmB)^83^ and type 6 secretion system (T6SS)^84^, however, no study up to date mentions MFS pumps as potential pyoverdine exporters. Thus, to the best of our knowledge, this study is the first report suggesting that an MFS efflux pump, the EmrAB-TolC-like system, might be involved in pyoverdine secretion by *Pseudomonas* spp., however, more research is required to confirm this hypothesis.

### Validation of RT-qPCR reference genes

According to the MIQE (Minimum Information for Publication of Quantitative Real-Time PCR Experiments) guidelines^35^, one of the most critical steps of a reliable RT-qPCR assay is the selection and validation of the appropriate reference genes (RGs). Many algorithms have been developed to avoid creating questionable datasets and assisting in selecting the correct RGs since the RT-qPCR was introduced^85^. While the existing selection algorithms for RGs such as geNorm, BestKeeper, or NormFinder may yield different results and are therefore imperfect tools, no better way to obtain reliable RGs has been developed. Hence, these algorithms are widely recognised as helpful in assessing the stability of the expression of bacterial genes^86^. Herein, we used the available algorithms to select the appropriate RGs to study gene expression in *P. donghuensis* P482. Out of ten initially chosen genes, three, namely *gyrB*, *rpoD* and *mrdA*, fulfilled the criteria of being most stably expressed under various experimental conditions (Fig. 2b,c), and therefore have been selected for gene expression study in *P. donghuensis*. This stands in contradiction to the gene expression analysis reported for another *P. donghuensis* strain, HYS^T^, where *rpoB* was used as a single reference gene^18^*.* The *rpoB* or 16S rRNA genes are still the default single reference genes used in bacterial gene expression studies^87–91^ despite proven instability of their expression under different conditions and in various species^39,92,93^. Although our results show that *rpoB* gene expression is relatively stable in *P. donghuensis* under the tested conditions (Fig. 2b,c), they also suggest that using only one RG is not sufficient for a given strain and under given conditions (Fig. 2d). The misinterpretation of gene expression data due to the use of one RG instead of two or more for normalisation has already been described in several studies^94,95^, as well as in MIQE guidelines^35^, alerting researchers to the shortcomings of this practice. As a result, in the last few years, there has been a growing number of published articles on microbiological research which focus in particular on the validation of RGs for RT-qPCR and the recognition of the MIQE guidelines^96–99^. Nevertheless, the thorough, careful selection of RGs is still overlooked in many RT-qPCR studies, not only in microbiological research but also in clinical studies, which ultimately leads to problems with proper diagnostics and therapies of patients, especially those with cancer^100^.

Hence, as a good practice and to promote reliable gene expression analysis, we present a complete study that led to establishing a set of genes that could be used as RGs in *Pseudomonas donghuensis*. To the best of our knowledge, it is the first report on the complete selection of RT-qPCR reference genes in this species.

### Carbon source-dependence of gene expression with reference to the antibacterial activity of P482

The yield of antimicrobial compounds produced by microorganisms like pseudomonads can be altered by nutritional conditions. Such influence of the growth medium has already been observed for pyrrolnitrin, phenazines and siderophores^29,57^. Furthermore, the *Pseudomonas* catabolite repression control system (Crc protein-dependent), which adapts bacterial cells to utilising the optimal carbon source in a given environment, has been involved in regulating the biosynthesis of pyocyanin, an antimicrobial pigment of *P. aeruginosa*^101^. In our study, diminished or lost antimicrobial activity of P482 mutants under different carbon source conditions was confronted with the expression level of the corresponding genes of P482 wt in analogous nutritional backgrounds. As expected, we did not observe a significant change in the expression of pyoverdine biosynthesis genes (namely, BV82_1009 and 3755, Fig. 3) in the presence of glucose or glycerol as a single carbon source. This remains in line with the antibiosis assays, as the KN1009 and KN3755 mutants exhibit a similar decline of antibacterial activity independently of the carbon source (Fig. 1a,b).

A pivotal role in regulating metabolism can be played by the most elementary nutrient, a carbon source, investigated in the presented study. Above all, glycerol, a simple polyol, has been the subject of many investigations that show its usefulness as a substrate for producing secondary metabolites in *Pseudomonas* spp.^102^. Research by Yao et al*.*^103^ suggests that glycerol utilised as a sole carbon source by *Pseudomonas chlororaphis* upregulates the expression of genes involved in phenazine-1-carboxamide biosynthesis as well as the yield of this antifungal compound. In the case of the gene expression of *P. donghuensis* P482 upon glycerol and another basic carbon source, glucose, our interest was focused on the 7-HT biosynthesis gene cluster. Inactivation of one of the genes from this cluster caused about 50% reduction in P482 antibacterial potential when glucose was the sole carbon source. However, when glycerol served as a carbon source, the inactivation of these genes had no impact on the antimicrobial activity of the given mutant in comparison to P482 wt (Fig. 1). The RT-qPCR results showed decreased expression of the studied genes necessary for 7-HT biosynthesis (loci: BV82_4705, BV82_4706 and BV82_4709) when glycerol served as the only carbon source, which was in contrast to what was observed for glucose. This supported our hypothesis that 7-HT is not the primary source of P482 antibacterial activity under such conditions, but another pathway has to be utilised to enable P482 to inhibit the growth of pathogens. It prompted us to analyse the UV–Vis absorption spectra of the post-culture supernatants of P482 wt and its mutants cultured in the M9 medium supplemented with glucose or glycerol (Supplementary Data Figure S7). The premise of this experiment was that both 7-HT and pyoverdine should be detected in the spectra as described by Jiang et al*.*^20^ and Chen et al.^18^. Nevertheless, these results did not confirm our interpretation of the gene expression data since the obtained absorption peaks pattern does not match the one described for *P. donghuensis* HYS^T^ cultured in MKB medium^18^. The inconsistency may be attributed to post-transcriptional and post-translational regulation phenomena and may imply that 7-HT is not produced under the tested conditions, but its biosynthesis pathway may still be involved in the antimicrobial activity of P482. It is also worth noting that in this spectrophotometric assay, no metabolites were detectable in the P482 culture supernatant until the fourth day of culture under the given conditions, which can be explained by the fact that many bacterial secondary metabolites are only produced after the culture enters the stationary growth phase and are accumulated over time^5^. Chen et al.^18^ also suggested the effect of medium composition on the 7-HT yield, which is high in King’s B medium (KBM), but low in LB medium. This takes place despite glycerol being the basic carbon source in KBM. Together with our results, this observation suggests that another nutrient present in KBM might be involved in the upregulation of 7-HT production in *P. donghuensis*. However, the data obtained in this study must be interpreted with caution. They should not be referred to other *P. donghuensis* strains because the nutritional regulation we observed might be strain-specific, as it was demonstrated for the production of 2,4-diacetylphloroglucinol and pyoluteorin in certain strains of *P. fluorescens* and *P. protegens*^6^ or as described by Poblete-Castro et al.^102^.

Surprisingly, however, regardless of the carbon source, glucose or glycerol, added to the medium, there was no significant increase in the expression level of BV82_4240 of the “cluster 17”. This gene seemed to be crucial for the P482 activity on glycerol, as the KN4240 mutant almost entirely lost its ability to inhibit the growth of pathogens (Fig. 1) under this condition. One possible explanation might be that the induction of high expression of the genes in this operon occurs only in the presence of the pathogen, as the potential pathogens’ signals can serve as activators of the synthesis of antimicrobials^104^. Such ecological issues should not be overlooked as they may constitute a further problem in applying biocontrol agents in the field. Another possible cause of the moderate discrepancy between RT-qPCR and antibiosis results for “cluster 17” could be, as with 7-HT, any type of post-transcriptional regulation.

Furthermore, we also measured the expression of the *gacA* gene in locus BV82_3318. It encodes for GacA protein—a component of a global regulatory system in *Pseudomonas* spp.^105^. This study shows that the expression of the *gacA* gene in P482 is 2.6-fold lower upon glycerol than glucose. Chen et al.^18^ proved that 7-HT biosynthesis in *P. donghuensis* HYS^T^ is positively regulated by a mechanism involving the Gac-Rsm system. Since our *gacA* expression analyses are consistent with the gene expression data obtained for 7-HT biosynthesis genes, they support the claim that the Gac-Rsm system modulates the 7-HT production.

In conclusion, the presented study provides new vital insights on *Pseudomonas donghuensis* antimicrobial activity and its regulation, as well as it introduces a novel genomic region, “cluster 17”, possibly involved in a production of an unknown antimicrobial. Moreover, it is noteworthy that a set of RT-qPCR reference genes for *P. donghuensis* P482 was established in the course of this investigation and the results of the gene expression study show that crucial changes in the expression of the 7-HT biosynthesis pathway genes occur due to the utilised carbon source (glucose or glycerol). This draws attention to the limitations of biological plant protection resulting from the nutritional conditions being an essential factor influencing gene expression and thus the activity of a given biocontrol agent. However, a considerable amount of research is still needed to fully understand the principles of antimicrobial activity and its regulation in *Pseudomonas donghuensis* P482 and other strains of this species in order to provide detailed information for their potential application as biocontrol agents.

## Materials and methods

### Strains and culture conditions

Bacterial strains and plasmids used in this study are presented in Table 4. All strains were routinely maintained in Miller’s Lysogeny Broth^106^ (LB, Novagen, Merck Group, Germany) or on plates with LB solidified with 1.5% (w/v) agar (LB-agar, Novagen, Merck Group, Germany). *P. donghuensis* P482 insertion mutants and every mutagenesis intermediate strain containing pKNOCK vector were cultured in LB or LB-agar supplemented with kanamycin (30 μg ml^−1^). Media for the growth of *E. coli* ST18 were supplemented with δ-aminolevulinic acid (5-ALA, Sigma-Aldrich, USA) (50 μg ml^−1^). During standard cultivation all strains were incubated overnight at 28 °C in stationary or shaking incubators (at 120 rpm shaking rate).

**Table 4:** Bacterial strains and plasmids used in the study.

Strain	Origin/features	References
***Pseudomonas* strains**		
*Pseudomonas donghuensis* P482	Tomato plant rhizosphere (Poland), wild type (wt)	Krzyżanowska et al*.*^9^
*Pseudomonas vranovensis* DSM16006^T^	Soil (Czech Republic); no antimicrobial activity (negative control strain)	Tvrzová et al*.*^107^
**Plant pathogenic strains**		
*Dickeya solani* IFB0102	Potato plant (Poland)	Sławiak et al*.*^108^
*Pseudomonas syringae* pv. s*yringae* Pss762	Apricot (Poland)	Kałużna et al*.*^109^
**Strains used in genetic engineering**		
*Escherichia coli* ST18	Donor strain for a biparental mating: *pro thi hsdR* + Tpr^R^ Smr^R^; chromosome:RP4-2 Tc::Mu-Kan::Tn7/λpir *ΔhemA*	Thoma et al*.*^110^
**Genetically modified strains**		
KN3318	*P. donghuensis* P482 with insertion of pKNOCK backbone in *gacA* gene locus	Krzyżanowska et al*.*^10^
KN4705, KN4706, KN4709	*P. donghuensis* P482 with insertion of pKNOCK backbone in respective loci (genes involved in a potential 7-HT production)	Krzyżanowska et al*.*^10^
KN1009, KN3755	*P. donghuensis* P482 with insertion of pKNOCK backbone in respective loci (genes involved in pyoverdine production)	Krzyżanowska et al*.*^10^
KN4240, KN4243	*P. donghuensis* P482 with insertion of pKNOCK backbone in respective loci (“cluster 17” genes)	This study
**Plasmids**		
pKNOCK-Km	2098 bp Km^R^ suicide vector for site directed mutagenesis	Alexeyev^111^
pKN4240	2492 bp Km^R^ pKNOCK-Km vector with 394 bp fragment of BV82_4240 gene (primers F_XbaI_KN4240 / R_XhoI_KN4240) in the XbaI_XhoI cloning site	This study
pKN4243	2490 bp Km^R^ pKNOCK-Km vector with 392 bp fragment of BV82_4243 gene (primers F_XbaI_KN4243 / R_XhoI_KN4243) in the XbaI_XhoI cloning site	This study

For the reference gene expression stability analyses and the RNA extraction, *P. donghuensis* P482 liquid cultures were carried out under different conditions and in various media as mentioned in Table 2 All strains were cultured overnight at 28 ℃ with shaking, unless otherwise stated. For RNA isolation, unless otherwise stated, the cultures were carried out till the late exponential/early stationary phase of growth as predicted with the P482 strain growth curve for each of the tested culture conditions (Supplementary Data Figure S5).

For the analysis of the gene expression under various carbon source conditions, M9 minimal medium prepared as described by Sambrook *et al.*^106^ was supplemented either with 0.4% glucose or 0.4% glycerol.

### Site-directed mutagenesis

The P482 mutants were obtained as described previously by Krzyżanowska et al*.*^10^. Briefly, amplicons (312–417 bp) being fragments of the genes to be inactivated were cloned into XbaI_XhoI cloning site of pKNOCK-Km suicide vector^111^. The 5-ALA auxotrophic *Escherichia coli* ST18^110^ donor competent cells were transformed with the obtained constructs named pKN4240 and pKN4243. The resulting *E. coli* [pKN4240] and *E. coli* [pKN4243] strains transferred the vectors by biparental mating with recipient *P. donghuensis* P482 cells. For this purpose, the cells obtained from the overnight cultures of the auxotrophic donor (appropriate *E. coli*) and the recipient (P482) were washed twice with fresh LB and resuspended in 0.5 ml LB medium. Equal volumes of each were mixed and then the mixed cells were harvested by centrifugation. The resulting pellet was resuspended in ca. 20 μl of LB medium and spotted on the centre of the LB-agar plate to enable the conjugation. After an overnight incubation at 37 ℃, the resulting macrocolony was scratched from the plate and suspended in 1 ml of sterile saline and three tenfold serial dilutions were prepared. Each suspension was plated on LB-agar medium supplemented with kanamycin (30 µg ml^−1^) as a selective factor for P482 pKNOCK vector recipients, but without 5-ALA (to inhibit the growth of ST18 donor strain). The colonies of the transconjugants obtained were then screened for the presence of the pKNOCK-Km insert with colony PCR using the pKNOCK-Km backbone primers. The insertion in the correct loci in the P482 genome was then confirmed by sequencing the fragment of the transconjugant genome starting with pKNOCK insert flanking region primers and mapping the insertion onto the P482 wild type strain genome. The sequencing was performed at Oligo.pl (Warsaw, Poland). The primers list and their sequences can be found in Supplementary Data Table S2.

### Direct antibacterial activity assay

Pre-culture conditions and suspensions preparation.

Pre-cultures of the tested strains were prepared to avoid nutritional contamination of media during the antibiosis assay. Five ml aliquots of liquid medium corresponding to the solid medium used for nutrient-dependent antibiosis tests were prepared. One colony of each of the strains: P482 wt, its KN mutants, *P. vranovensis* DSM16006 (a negative control strain) and *D. solani* IFB0102 or *P. syringae* pv. *syringae* Pss672 (pathogens) was used to inoculate the appropriate liquid medium and the cultures were incubated at 28 °C for 20 h (media with glucose) or 44 h (media with glycerol). The cultures’ turbidity was subsequently measured and adjusted to 4 McFarland units (McF) for pathogenic strains and 12 McF for P482 wt, P482 KN mutants and a negative control strain, DSM16006.

### Experimental conditions

For direct antibiosis assay, the M9 minimal medium solidified with 1.5% w/v agar was used. For carbon source dependency of the antimicrobial activity this medium was supplemented with 0.4% (w/v) glucose or 0.4% (v/v) glycerol.

One hundred μl of *D. solani* IFB0102 or *P. syringae* pv. *syringae* Pss762 (4 McF) was spread on the appropriate M9 plate and subsequently 2 μl drops of suspensions (12 McF) of P482 wild type, tested mutants and control strain were spotted. Plates were incubated at 28 °C for 20–44 h, until the pathogen growth inhibition zones around tested strains’ spots were visible and measurable (example in Supplementary Data Figure S1). The diameters of the growth inhibition zones were measured (measurements in Supplementary Data Table S1). The experiment was performed in 3 biological replicates. For the data analysis the results were normalised and shown as a percentage of the growth inhibition zone caused by P482 wt which was measured in the same biological replicate of the sample (the same plate). A basic statistical analysis of the results was conducted which consisted of calculation of the growth inhibition mean values from the replicates and the result variability was tested by calculating standard deviations for each sample.

### *In silico* gene and protein sequence analysis

For prediction of the operon’s organisation in the “cluster 17” the Operon Mapper tool (https://biocomputo.ibt.unam.mx/operon_mapper)^37^ was applied. KEGG database search (http://www.genome.ad.jp/kegg)^112^ was performed to find functional orthologs of the genes in “cluster 17” and InterPro (https://www.ebi.ac.uk/interpro/)^113^ and NCBI (https://www.ncbi.nlm.nih.gov/) databases were used to analyse and predict domains and motifs of proteins encoded by “cluster 17” genes. In order to identify the functional categories of the products of these genes’ eggNOG-mapper online tool^114^ was utilised.

### RNA isolation and reverse transcription (RT)

Total RNA isolation from bacterial cultures was carried out as instructed in the manufacturer’s protocol with RNeasy Mini Kit (Qiagen, Germany). The P482 was cultured in various conditions (Table 2) in 3 biological replicates. Approximately 2.5 × 10^8^ bacterial cells per single isolation were used. The bacterial cells were harvested by centrifugation and suspended in 500 µl of sterile saline. To prevent RNA degradation, 1 ml of RNAprotect Bacteria Reagent (Qiagen, Germany) was immediately added to each sample. After the RNA isolation procedure, genomic DNA (gDNA) contamination of each sample was confirmed by subjecting 1 µl of an RNA sample obtained to a 30-cycle PCR reaction with *rpoB* primers designed for qPCR (Supplementary Data Table S2) and the RNA samples were stored at -80 °C. The concentration and quality of the RNA samples were measured with NanoDrop 2000 (Thermo Scientific, USA). The RNA concentration in the samples varied in the range of 100–600 ng per µl and was adjusted for a single reverse transcription reaction.

Reverse transcription (RT) of RNA to cDNA was performed as instructed in the manufacturer’s protocol with iScript gDNA Clear cDNA Synthesis Kit (Bio-Rad, USA) and an optional DNA digestion was performed prior to the RT step to ensure lack of gDNA contamination. Random hexamers were utilised as primers. Total amount of RNA used per single reaction was adjusted to 500 ng. After the RT procedure the samples, were immediately subjected to qPCR or stored at -20 °C up to 2 months.

### Quantitative PCR

Primers (Supplementary Data Table S2) (Sigma-Aldrich, Merck Group, Germany) were designed with PerlPrimer^115^ and Primer3^116^ tools and tested for their specificity in silico with the use of BLAST tool. The qPCR assays were carried out using the CFX96 thermocycler coupled with CFX Maestro software (Bio-Rad, USA). The PCR conditions were 95 °C for 5 min followed by 40 cycles of 95 °C for 15 s, 60 °C for 30 s. Each PCR run was followed by a melting curve step (65–95 °C, increment: 0.5 °C/5 s). The reaction mixture (total volume: 15 µl) consisted of Sso Advanced Universal SYBR Green Supermix (Bio-Rad, USA), forward and reverse primer at a final concentration of 200 nM and 5 µl of the sample cDNA template (1:3 H_2_O diluted post-RT mixture). Each reaction was run in 2 technical replicates. The results were included in the analysis when the quantitation cycle (C_q_) difference between the duplicates (replicate variability) was lower than 0.3 cycle and no template controls (NTCs) in each run for each pair of primers resulted in C_q_ values > 37 (typically NTCs yielded no signal). Inter-run calibrating sample was included in each run. Quality parameters of the assay were validated prior to RT-qPCR experiments. These included assessments of primers efficiency, assay specificity and linearity. Primers’ efficiency and assay linearity were assessed by obtaining 7-point standard curves^117^ for each primer pair with the using of 10-fold dilutions of post-PCR amplicons that served as corresponding templates. Primer specificity was confirmed with both observation of the PCR product on gel electrophoresis and the melting curves.

### Analysis of expression stability and the optimal number of reference genes

Expression of 9 candidate RGs was tested under 12 culture conditions (Table 2). The expression stability was assessed with the use of geNorm algorithm^94^ integrated into the qbase + software, version 3.2^118^ (Biogazelle NV, Belgium – www.qbaseplus.com). Additionally, the expression stability was assessed using the RefFinder tool^119^ (www.heartcure.com.au/reffinder/). RefFinder is a comprehensive web tool that employs four different algorithms (ΔCT^120^, BestKeeper^121^, NormFinder^122^ and previously mentioned geNorm) that calculate reference gene stability using raw C_q_ (threshold cycle) data. Since there’s no other factor (eg. primer efficiency or inter-run calibration) taken into consideration in these calculations, they were only performed as an additional confirmation of qbase + incorporated geNorm results and only the short synopsis of the RefFinder results is included in the text body.

### Analysis of gene expression

Gene expression analysis was carried out with the use of qbase + software, version 3.2 (Biogazelle NV, Belgium) by applying the general ∆∆C_t_ approach with normalisation to reference genes^118^. The values of normalised relative quantity (NRQ) attained were calibrated in accordance with values acquired for the used inter-run calibrator sample to obtain calibrated NRQ (CNRQ) values which represented the relative expression values and have subsequently been subjected to statistical analysis. The statistical analysis was also carried out in qbase + software. The mean CNRQ values obtained for each tested gene in various conditions were subjected to comparison with the use of two-tailed Student’s t-test with correction for multiple testing.

## Supplementary Information

Supplementary Information.

## Data Availability

All data generated or analysed during this study are included in this published article (and its Supplementary Information files).
