# Supplementary material for: The carbon source-dependent pattern of antimicrobial activity and gene expression in Pseudomonas donghuensis P482
Source: Sci Rep. 2021 May 26;11:10994. doi: 10.1038/s41598-021-90488-w (PMC8154892; doi:10.1038/s41598-021-90488-w)
Supplement: Supplementary file 1 — Supplementary Information. [file 41598_2021_90488_MOESM1_ESM.pdf]

# SUPPLEMENTARY DATA

## The carbon source-dependent pattern of antimicrobial activity and gene expression in *Pseudomonas donghuensis* P482

**Marta Matuszewska, Tomasz Maciąg, Magdalena Rajewska, Aldona Wierzbicka and Sylwia Jafra***

Laboratory of Plant Microbiology, Intercollegiate Faculty of Biotechnology of University of Gdansk and Medical University of Gdansk, University of Gdansk, Gdansk, Poland.

*Correspondence and requests for materials should be addressed to S.J. (email: sylwia.jafra@ug.edu.pl)

## Supplementary Figures

**Figure S1. Antibiosis assay for P482 wt and its mutants against *Dickeya solani* IFB102 strain on the minimal M9 medium with either 0.4% glucose or 0.4% glycerol**. The photographs show representative images of the inhibition zones observed in one biological replicate (each column shows results from a single plate). The inhibition zone obtained for P482 wt served as a reference against which the diameters of the inhibition zones produced by the mutants were compared.

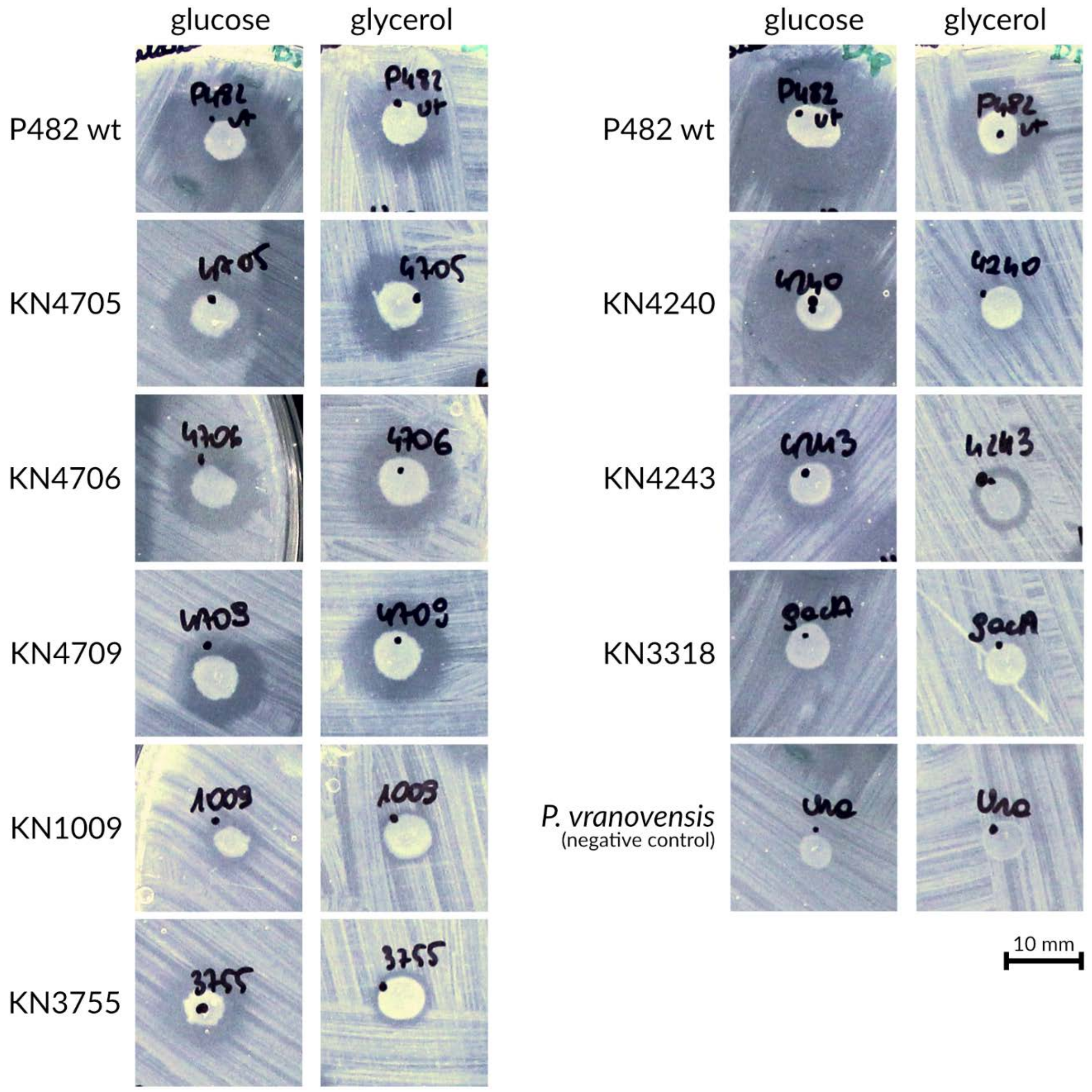

**Figure S2. Graphic representation of ORF organization in the P482 gene "cluster 17".** Grey arrows represent identified promoter sequences. Asterisks mark genes targeted in pKNOCK site-directed mutagenesis. The letters above the ORFs represent functional category of the product of each gene as in COG (Clusters of Orthologous Genes) classification assigned by the eggNOG-mapper tool (Huerta-Cepas *et al.*, 2017): Q – secondary metabolites biosynthesis, transport and catabolism, H – coenzyme transport and metabolism, I – lipid transport and metabolism, S – function unknown, V – defense mechanisms, E – amino acid transport and metabolism, G – carbohydrate transport and metabolism, P – inorganic ion transport and metabolism, M – cell wall/membrane/envelope biogenesis, U – intracellular trafficking, secretion, and vesicular transport. Information concerning gene annotation can be found in Table 1.

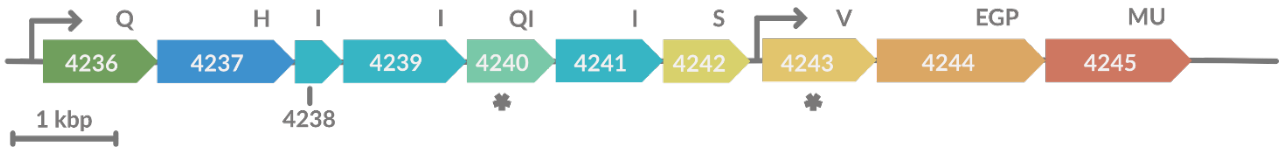


**Figure S3. Growth inhibition of *Dickeya solani* IFB0102 and *Pseudomonas syringae* pv. *syringae* Pss762 by *Pseudomonas donghuensis* P482 wt and its mutants on LB-agar medium**. The test was conducted according to the antibiosis assay on the M9 medium described in the Materials and Methods section. The bars represent the percentage of the growth inhibition zone obtained for the P482 wt strain under given condition. Error bars represent standard deviation (n = 8).

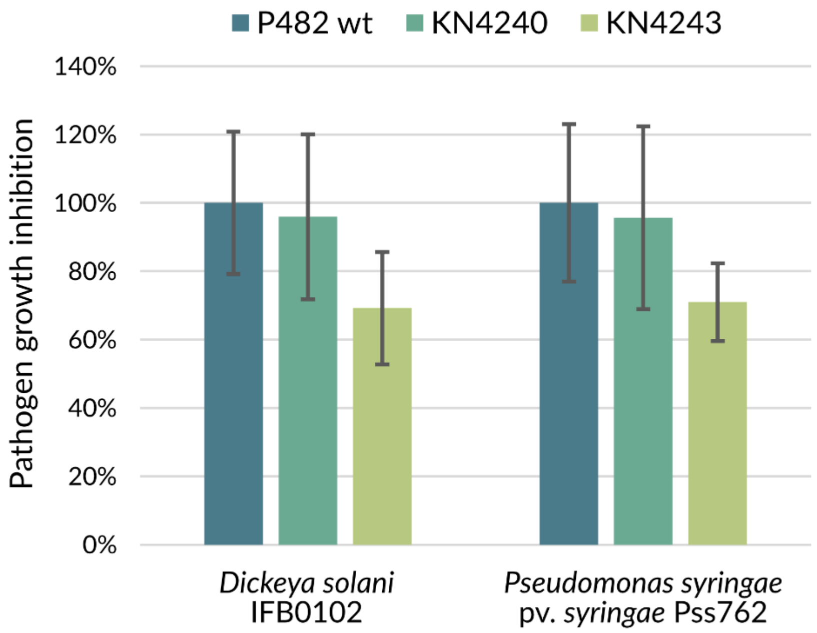

**Figure S4.** Results of analyses of the stability of r**eference genes as calculated by RefFinder with four algorithms: (a) ΔCT, (b) NormFinder, (c) BestKeeper, (d) geNorm**. The lowest values obtained with each of the algorithms indicate the most stable reference genes. The overall ranking based on geometric mean of the gene ranks from each of the algorithms can be found in Figure 2b.

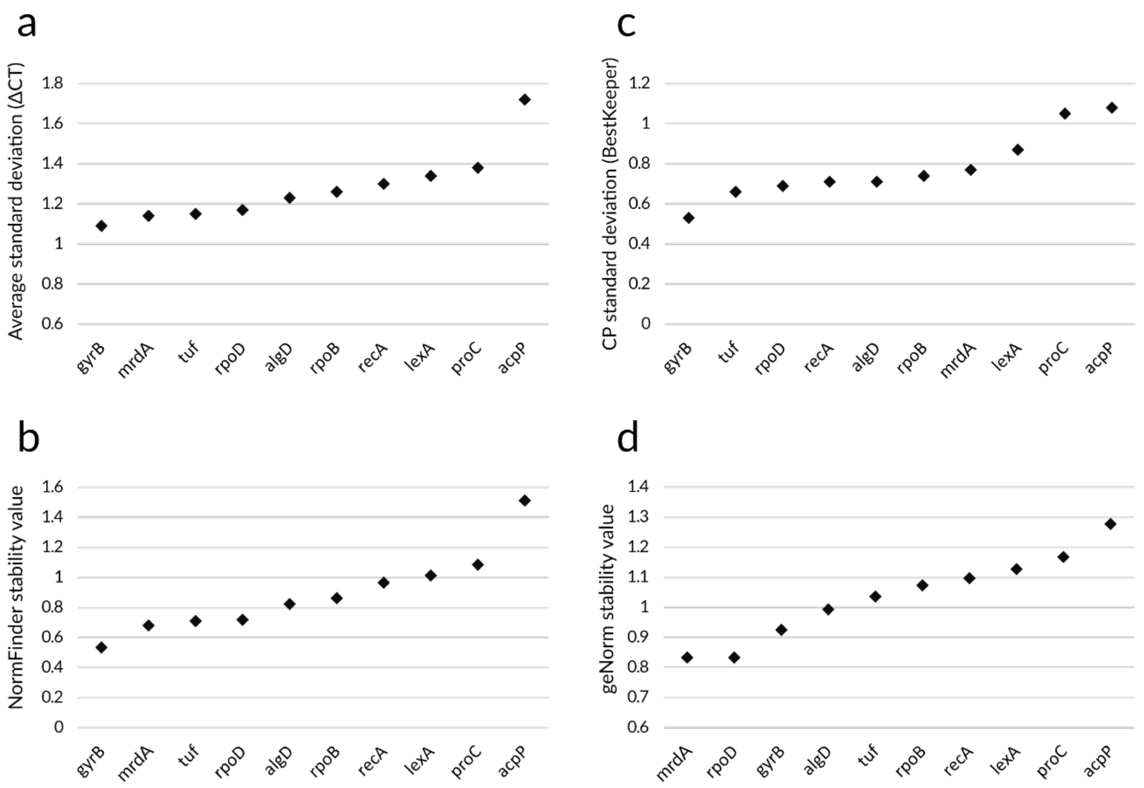

**Figure S5. Growth curves for *Pseudomonas donghuensis* P482 wild type strain and its mutants were determined prior to all performed tests**. The presented growth curves were used to select the endpoints for cultures used in gene expression experiments. To obtain the growth curves the strains were pre-cultured for 24 h at 28°C in the same type of medium that was later used in the growth curve assay. The optical density of precultures was measured and adjusted so that 10 µl of a preculture (1 McF) were added to 200 µl of a fresh M9 medium supplemented with (a) 0.4% glucose or (b) 0.4% glycerol. The 96-well plate with test cultures was placed in the chamber of Epoch™ Microplate Spectrophotometer (BioTek, USA). The protocol comprised 44 h of culture (with shaking) at 28°C with 600 nm absorbance measurements (OD600) every 20 minutes; 4 replicates of each sample were measured. Mean value for each readout was used to produce growth curves of each strain under given conditions.

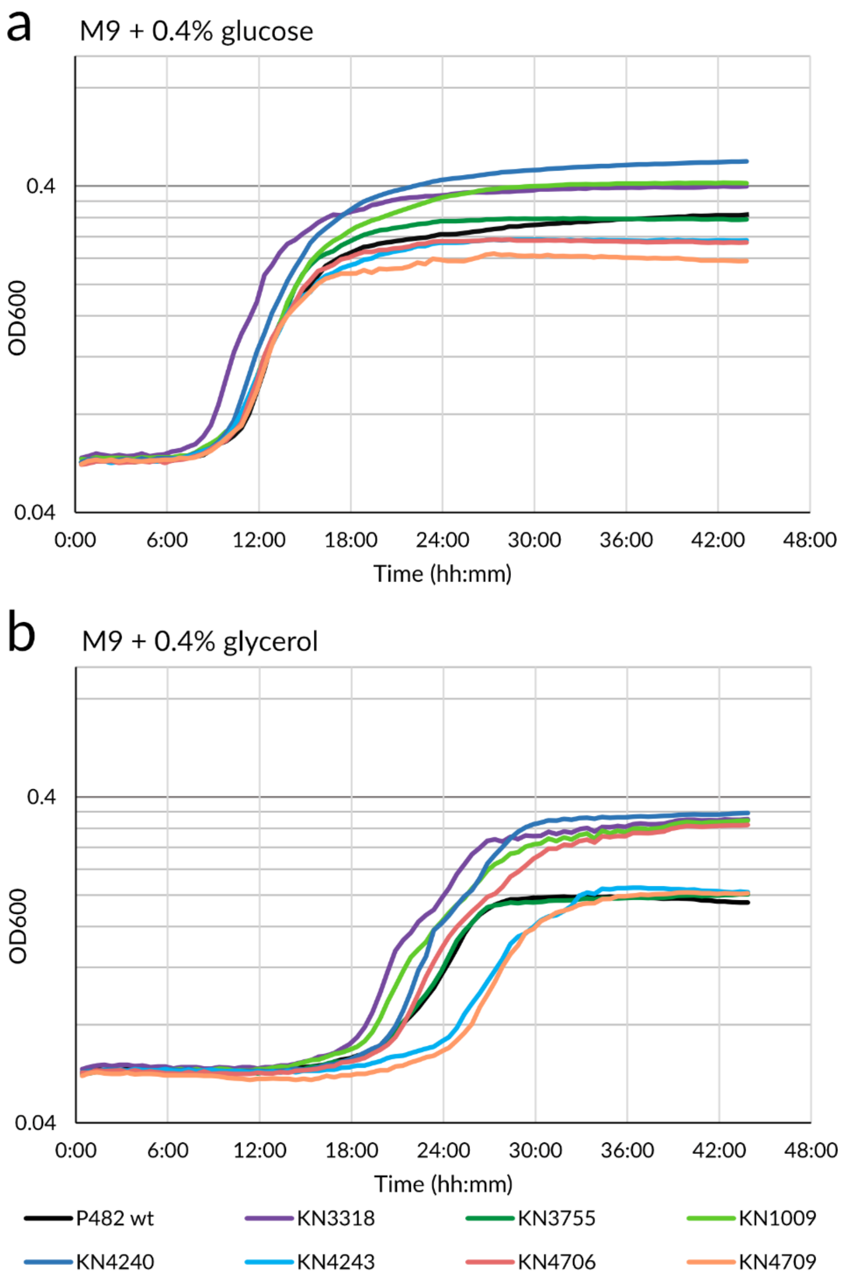

**Figure S6. Melting curves of qPCR P482 targets and reference genes.** All qPCR reactions in the study were followed by a melt curve step (65-95°C, increment: 0.5°C/5 sec). Each plot is a combination of peaks obtained for one target in at least 10 reactions using different P482 cDNA samples as matrix.

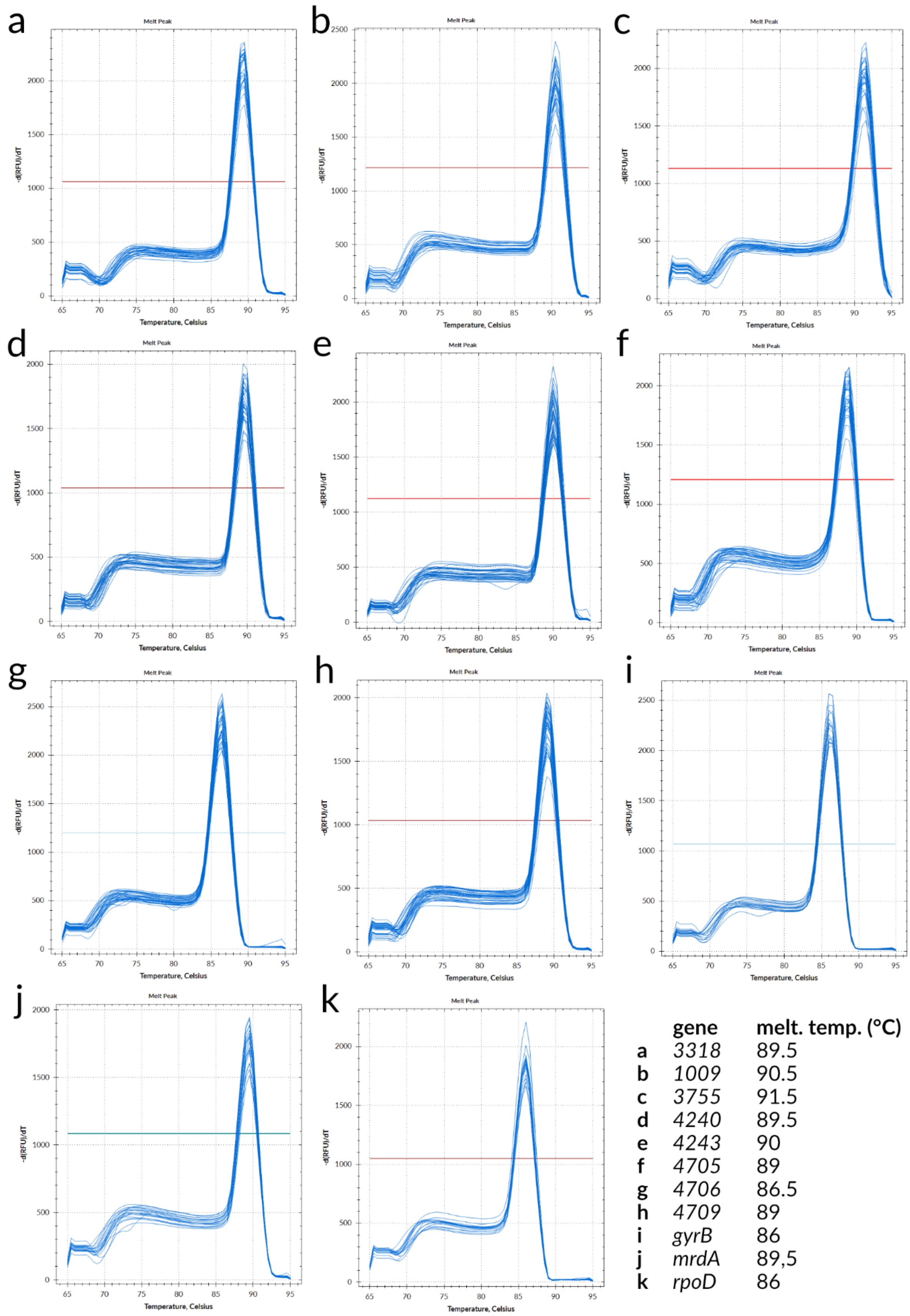


| | gene | melt. temp. (°C) |
|---|---|---|
| a | *3318* | 89.5 |
| b | *1009* | 90.5 |
| c | *3755* | 91.5 |
| d | *4240* | 89.5 |
| e | *4243* | 90 |
| f | *4705* | 89 |
| g | *4706* | 86.5 |
| h | *4709* | 89 |
| i | *gyrB* | 86 |
| j | *mrdA* | 89,5 |
| k | *rpoD* | 86 |

**Figure S7. (a) UV-visible absorption spectra of the culture supernatants of P482 wt and its mutants.** To obtain the spectra, P482 wt and its mutants were cultured in liquid M9-glucose (black line, crosses) or M9-glycerol (grey line, dots) medium for 96 hours. Supernatants were collected after the centrifugation of cultures and their UV-Vis absorbance spectra were measured in the range of 200-550 nm (Epoch™ Microplate Spectrophotometer, BioTek, USA). The presence of produced iron chelators (7-HT and pyoverdine) in each supernatant was determined by the observation of characteristic absorption peaks: one peak at approximately 405 nm for pyoverdine and two peaks at approximately 330 and 392 nm for 7-HT (Jiang *et al.*, 2016). **(b) Representative photograph of the visible fluorescence of M9-glucose culture supernatants of P482 and mutants.** The fluorescent pigment is pyoverdine, visualised under a UV lamp (365 nm wavelength). The carbon source in the medium (glucose or glycerol) had no effect on the visible fluorescence effect of the supernatants.

a

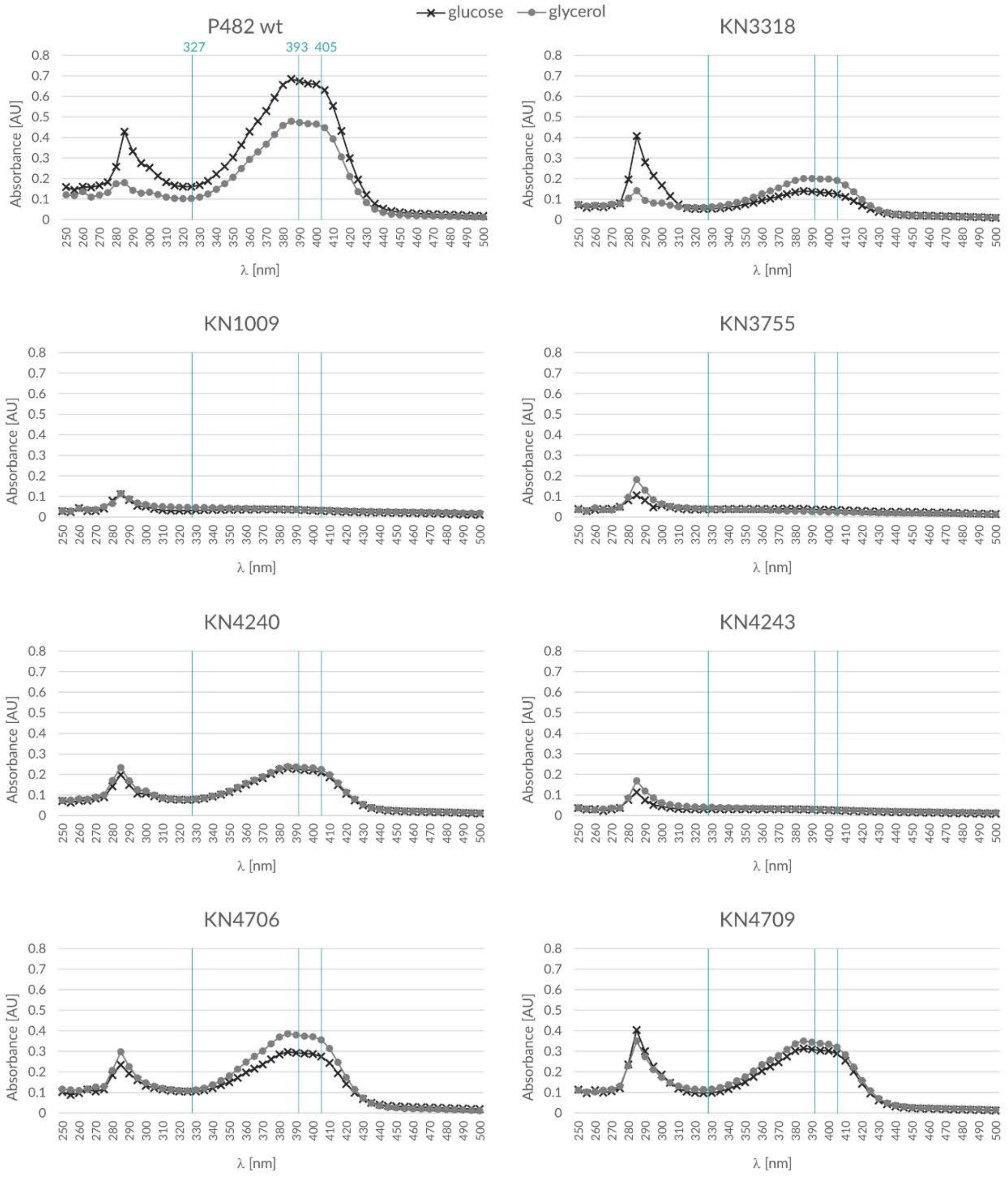


b

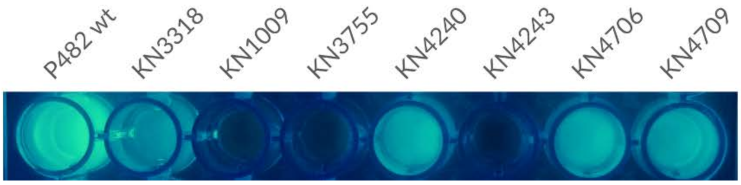

## Supplementary Tables

**Table S1. Diameters of the growth inhibition zones of *D. solani* IFB0102 and *P. syringae* pv. *syringae* caused by *P. donghuensis* P482 and its mutants tested on M9 agar medium with glucose or glycerol as a sole carbon source.** The mean zone diameter was calculated as the arithmetic means of the measured diameters under the given conditions. The percentage of the zone diameter caused by P482 wt was always first calculated concerning the P482 wt grown on the same plate as the given mutant (exact biological replicate). The mean percentages and standard deviations were calculated subsequently. These results are visualised as charts in Figure 1 and with photographs in Supplementary Figure S1.

| Strain | *D. solani* IFB0102 growth inhibition | | | | | |
|---|---|---|---|---|---|---|
| | glucose | | | glycerol | | |
| | mean zone (mm) | mean % of P482 wt zone | st. dev | mean zone (mm) | mean % of P482 wt zone | st. dev (%) |
| **P482 wt** | 12.7 | 100.00% | 3.09% | 5.8 | 100.00% | 12.69% |
| **KN4705** | 5.8 | 45.25% | 2.63% | 5.3 | 97.44% | 10.94% |
| **KN4706** | 5.8 | 45.25% | 2.63% | 5.7 | 103.33% | 5.77% |
| **KN4709** | 5.8 | 45.07% | 6.02% | 5.3 | 96.67% | 5.77% |
| **KN1009** | 2.3 | 17.54% | 8.71% | 0.8 | 15.13% | 5.00% |
| **KN3755** | 5.5 | 42.47% | 5.05% | 1.2 | 21.03% | 1.78% |
| **KN4240** | 12.0 | 95.83% | 4.17% | 0.0 | 0.00% | 0.00% |
| **KN4243** | 5.2 | 41.51% | 4.40% | 1.5 | 24.01% | 6.35% |
| **KN3318** | 0.0 | 0.00% | 0.00% | 0.0 | 0.00% | 0.00% |
| | *P. syringae* pv. *syringae* Pss762 growth inhibition | | | | | |
| | glucose | | | glycerol | | |
| | mean zone (mm) | mean % of P482 wt zone | st. dev (%) | mean zone (mm) | mean % of P482 wt zone | st. dev (%) |
| **P482 wt** | 13.0 | 100.00% | 5.09% | 5.3 | 100.00% | 7.16% |
| **KN4705** | 6.8 | 51.16% | 6.66% | 4.7 | 85.45% | 12.61% |
| **KN4706** | 6.5 | 48.08% | 8.43% | 5.3 | 97.62% | 4.12% |
| **KN4709** | 4.8 | 35.66% | 4.28% | 5.3 | 97.62% | 4.12% |
| **KN1009** | 1.5 | 11.25% | 10.52% | 1.3 | 23.60% | 4.45% |
| **KN3755** | 7.3 | 54.28% | 0.51% | 1.7 | 30.63% | 2.44% |
| **KN4240** | 12.0 | 96.85% | 17.79% | 0.5 | 9.76% | 1.17% |
| **KN4243** | 5.0 | 40.48% | 6.77% | 1.3 | 26.26% | 7.63% |
| **KN3318** | 3.2 | 23.35% | 3.59% | 3.7 | 66.03% | 5.74% |

**Table S2. Primers designed and used in the study.**

| **Target gene/sequence** (GenBank locus tag) | **Encoded protein/Description** | **Primers** | **Primer sequences** [1] | **Amplicon length (bp)** |
|---|---|---|---|---|
| ***RT-qPCR reference gene selection primers*** | | | | |
| *rpoB* (BV82_1963) | RpoB, the β subunit of bacterial RNA polymerase | P482_rpoB_F<br>P482_rpoB_R | 5’ TTCACCACGATCCACATCCA<br>5’ CTTCAGCACCCACATAAACGA | 147 |
| *rpoD* (BV82_1895) | RpoD ($\sigma^{70}$), a “housekeeping” sigma factor involved in initiation of transcription | P482_rpoD _F<br>P482_rpoD_R | 5’ CCACGACGGTATTCGAACTT<br>5’ CGTGCCAAGAAAGAAATGGT | 152 |
| *gyrB* (BV82_2296) | DNA gyrase subunit B | P482_gyrB_F<br>P482_gyrB_R | 5’ ATCGACAAGCTGCGCTATCA<br>5’ CGGCTGAGCGATGTAGATGT | 144 |
| *mrdA/pbp-2* (BV82_4935) | Penicillin binding protein 2 | P482_mrdA_F<br>P482_mrdA_R | 5’ CTTGATCGCTACCACCTGAG<br>5’vCAAGTCGGACTGGAACAAGG | 138 |
| *recA* (BV82_0583) | Recombinase A | P482_recA_F<br>P482_recA_R | 5’ CTACCTGTGCCTTCGTTGAC<br>5’ CAGCATGTCGGTGATTTCCA | 133 |
| *lexA* (BV82_3405) | Transcriptional repressor LexA | P482_lexA_F<br>P482_lexA_R | 5’ CGTTCAAAGCGCTTGATGGT<br>5’ CCGACTACCTGCTCAAGGTG | 153 |
| *tuf* (BV82_0583) | Elongation factor Tu | P482_tuf_F<br>P482_tuf_R | 5’ CCCTACATCGTGGTCTTCCT<br>5’ GAACCAATGATGATCGGAGTG | 136 |
| *algD* (BV82_3321) | GDP-mannose 6-dehydrogenase | P482_algD_F<br>P482_algD_R | 5’ GAATATCAGCATCTTTGGATTGGG<br>5’ CTGGTTGATCATGTCGATCTTGG | 120 |
| *proC* (BV82_2548) | Pyrroline-5-carboxylate reductase | P482_proC_F<br>P482_proC_R | 5’ CAATCGAGACCACCAGTTGC<br>5’ GCATCGAGATGTTCGCAGAC | 140 |
| *acpP* (BV82_4055)[2] | Acyl carrier protein | P482_acpP_F<br>P482_acpP_R | 5’ GTCGATAGCAGCTTGAACAG<br>5’ AGAAGTGAAGAACGAATCTTCC | 150 |
| ***Primers for RT-qPCR targets of interest*** | | | | |
| BV82_1009 | A putative NRPS for chromophore synthesis (Krzyżanowska *et al.*, 2016) | P482_1009_F<br>P482_1009_R | 5’ CAATGGCAGAGCGAATAC<br>5’ CCGTCCTCAACCAGTAAG | 140 |

| | | | | |
|---|---|---|---|---|
| BV82_3755 | Partially similar to the PdvD pyoverdine synthase of *P.aeruginosa* (Krzyżanowska *et al.*, 2016) | P482_3755_F<br>P482_3755_R | 5’ AAAGCGAACGACATGAACAG<br>5’ CTGGCGTACCTGATCTACAC | 151 |
| BV82_4705 | Bacterial regulatory s, tetR family protein (7-HT biosynthesis cluster) | P482_4705_F<br>P482_4705_R | 5’ CTTCCTCAGCAACGAATCCA<br>5’ GACCATCGAGGTGATCATCTG | 150 |
| BV82_4706 | HpcH/HpaI aldolase/citrate lyase family protein (7-HT biosynthesis cluster) | P482_4706_F<br>P482_4706_R | 5’ AGGGCAGTTTCGATATTCGG<br>5’ GTCATGATGTCCAAGGTCGAG | 127 |
| BV82_4709 | Acyl-CoA dehydrogenase, C-terminal domain protein | P482_4709_F<br>P482_4709_R | 5’ GATTTCATGCTGATCGTGACC<br>5’ AGGTTGTCGAAATACAGCGG | 157 |
| BV82_4240 | SDR family NAD(P)-dependent oxidoreductase | P482_4240_F<br>P482_4240_R | 5’ AACCTGATGAACAAGACCGTG<br>5’ TAGAAGGCATCCAAGTGCAG | 145 |
| BV82_4243 | Efflux transporter, RND family, MFP subunit | P482_4243_F<br>P482_4243_R | 5’ CGCCAATTTCAAGGAAACCC<br>5’ CTACCTTGGTGAAGTTGCCG | 178 |
| *gacA* (BV82_3318) | GacA, a response regulator of the two-component GacS/GacA system | P482_3318_F<br>P482_3318_R | 5’ CAATTTGCGGGCTGATGTAG<br>5’ GGTGACCGTCTGTGAAGAAG | 148 |
| ***Primers used in site-directed mutagenesis*[3]** | | | | |
| BV82_4240 | BV82_4240 gene fragment for cloning into pKNOCK vector | F_XbaI_KN4240<br>R_XhoI_KN4240 | 5’ ATTATTCTAGATTGGATGCCTTCTACCTGCT<br>5’ AATTTCTCGAGACGTCTGAGTAGGCGCTCAT | 394 |
| BV82_4243 | BV82_4243 gene fragment for cloning into pKNOCK vector | F_XbaI_KN4243<br>R_XhoI_KN4243 | 5’ ATTATTCTAGAAACAGGGCGAACTGCTCTAC<br>5’ AATTTCTCGAGGCGGTATTCCTCGACTTTCA | 392 |
| pKNOCK vector[4] | pKNOCK-Km backbone | F_pKNOCK_backbone[5]<br>R_pKNOCK_backbone[5] | 5’ GGTGCCCTGAATGAACTCCA<br>5’ AAAAGCGGCCATTTTCCAC | - |
| pKNOCK vector[4] | pKNOCK insert flanking region | F_outof_pKNOCK[5]<br>R_outof_pKNOCK | 5’ CACGTACTAAGCTCTCATGTTTGAACA<br>5’ CTGGCAATTCCGGTTCGCT | - |

[1] Synthesis of oligonucleotides was outsourced to Sigma-Aldrich (USA).
[2] *acpP* gene was excluded from qbase+ geNorm analysis.[3] Sequences recognised by restriction enzymes (XbaI and XhoI) are underlined
[4] Alexeyev, 1999
[5] Krzyżanowska *et al.*, 2016

**Table S3. Ranking of the RefFinder comprehensive reference gene stability.** The comprehensive stability value (CSV) in the RefFinder is calculated as a geometric mean of 4 ranking values for each gene obtained in each of the 4 algorithms available in RefFinder: delta CT, BestKeeper, NormFinder and geNorm (results of each calculation are presented in Figure S5). Underlined are the names of the genes selected as reference genes in this RT-qPCR study.

| **Stability ranking** | 1 | 2 | 3 | 4 | 5 | 6 | 7 | 8 | 9 | 10 |
|---|---|---|---|---|---|---|---|---|---|---|
| **Gene name** | <u>*gyrB*</u> | <u>*mrdA*</u> | <u>*rpoD*</u> | *tuf* | *algD* | *rpoB* | *recA* | *lexA* | *proC* | *acpP* |
| **RefFinder CSV** | 1.32 | 2.3 | 2.63 | 3.08 | 4.73 | 6 | 6.09 | 8 | 9 | 10 |

**Table S4. Primer pair qPCR efficiencies and slopes calculated from standard curves for both test and reference targets.**

| **Target** | **E*** | **E (SE)**** | **$R^2$** | **Slope** | **Slope error** |
|---|---|---|---|---|---|
| *BV82_1009* | 2.00 | 0.05 | 0.997 | -3.31 | 0.11 |
| *BV82_3318* | 1.95 | 0.01 | 0.999 | -3.46 | 0.04 |
| *BV82_3755* | 2.05 | 0.02 | 0.999 | -3.20 | 0.05 |
| *BV82_4240* | 2.06 | 0.02 | 0.998 | -3.18 | 0.05 |
| *BV82_4243* | 1.97 | 0.05 | 0.991 | -3.40 | 0.13 |
| *BV82_4705* | 1.97 | 0.05 | 0.979 | -3.39 | 0.14 |
| *BV82_4706* | 2.03 | 0.02 | 0.999 | -3.25 | 0.05 |
| *BV82_4709* | 1.95 | 0.02 | 0.998 | -3.45 | 0.06 |
| *gyrB* | 1.96 | 0.08 | 0.981 | -3.41 | 0.21 |
| *mrdA* | 2.11 | 0.05 | 0.994 | -3.07 | 0.10 |
| *rpoD* | 1.96 | 0.02 | 0.999 | -3.42 | 0.05 |

*E – primer pair efficiency calculated as in $E = 10^{-1/slope}$ (Rasmussen, 2001)
**E(SE) - efficiency standard error

**Table S5. Raw data of P482 relative gene expression under given conditions**. Data presented separately for each analysed gene. CNRQ = Calibrated Normalized Relative Quantities, SE = standard error (calculated with qbase+ software).

| | Relative gene expression (CNRQ) | | | | | | | | | | | | | |
|---|---|---|---|---|---|---|---|---|---|---|---|---|---|---|
| **Sample ID (conditions)** | **10% TSB** | | | | | | **M9 glucose** | | | | | **M9 glycerol** | | |
| **Rep*** | **1** | **2** | **3** | **4** | **5** | **6** | **1** | **2** | **3** | **4** | **5** | **1** | **2** | **3** |
| **3318** | nd | nd | nd | 0.243 | 0.146 | 0.183 | nd | 0.201 | nd | 0.040 | 0.019 | -0.260 | -0.353 | -0.370 |
| **3318 (SE)** | nd | nd | nd | 0.031 | 0.018 | 0.024 | nd | 0.283 | nd | 0.107 | 0.056 | 0.081 | 0.064 | 0.076 |
| **1009** | 0.565 | 0.980 | 0.948 | nd | nd | nd | 0.525 | 0.465 | nd | 0.215 | nd | 0.257 | -0.346 | -0.072 |
| **1009 (SE)** | 0.038 | 0.049 | 0.068 | nd | nd | nd | 0.040 | 0.079 | nd | 0.079 | nd | 0.078 | 0.059 | 0.084 |
| **3755** | 0.791 | 0.946 | 0.947 | nd | nd | nd | nd | 0.297 | nd | 0.517 | 0.138 | 0.225 | -0.143 | -0.059 |
| **3755 (SE)** | 0.140 | 0.042 | 0.040 | nd | nd | Nd | nd | 0.020 | nd | 0.120 | 0.040 | 0.095 | 0.060 | 0.083 |
| **4240** | nd | nd | nd | 0.465 | 0.429 | 0.501 | nd | nd | -0.107 | 0.167 | -0.008 | -0.077 | -0.002 | -0.214 |
| **4240 (SE)** | nd | nd | nd | 0.021 | 0.019 | 0.031 | nd | nd | 0.101 | 0.075 | 0.033 | 0.077 | 0.073 | 0.078 |
| **4243** | nd | nd | nd | 0.713 | 0.579 | 0.644 | nd | nd | -0.426 | -0.195 | -0.399 | 0.081 | -0.204 | -0.263 |
| **4243 (SE)** | nd | nd | nd | 0.031 | 0.040 | 0.041 | nd | nd | 0.112 | 0.087 | 0.100 | 0.120 | 0.083 | 0.103 |
| **4705** | nd | nd | nd | 1.370 | 0.829 | 0.817 | nd | nd | 0.385 | 0.257 | 0.425 | -0.430 | -0.141 | -0.278 |
| **4705 (SE)** | nd | nd | nd | 0.049 | 0.038 | 0.067 | nd | nd | 0.100 | 0.039 | 0.033 | 0.089 | 0.071 | 0.103 |
| **4706** | nd | nd | nd | 1.527 | 1.390 | 1.504 | nd | nd | 0.684 | 0.521 | 0.701 | -0.598 | -0.315 | -0.624 |
| **4706 (SE)** | nd | nd | nd | 0.024 | 0.022 | 0.031 | nd | nd | 0.151 | 0.067 | 0.034 | 0.082 | 0.062 | 0.078 |
| **4709** | nd | nd | nd | 1.879 | 1.776 | 1.948 | nd | nd | 0.712 | 0.568 | 0.934 | -0.890 | -0.806 | -0.893 |
| **4709 (SE)** | nd | nd | nd | 0.036 | 0.040 | 0.049 | nd | nd | 0.148 | 0.082 | 0.034 | 0.091 | 0.070 | 0.080 |

* biological replicate ; nd = given biological replicate was not used in RT-qPCR for the given gene expression analysis

**References:**


1. Alexeyev, M. F. The pKNOCK Series of Broad-Host-Range Mobilizable Suicide Vectors for Gene Knockout and Targeted DNA Insertion into the Chromosome of Gram-Negative Bacteria. *Biotechniques* **26**, 824–828 (1999).
2. Huerta-Cepas, J. *et al.* Fast genome-wide functional annotation through orthology assignment by eggNOG-mapper. *Mol. Biol. Evol.* **34**, 2115–2122 (2017).
3. Jiang, Z., Chen, M., Yu, X. & Xie, Z. 7-Hydroxytropolone produced and utilized as an iron-scavenger by *Pseudomonas donghuensis*. *BioMetals* **29**, 817–826 (2016).
4. Krzyżanowska, D. M. *et al.* When genome-based approach meets the 'Old but Good': Revealing genes involved in the antibacterial activity of *Pseudomonas* sp. P482 against soft rot pathogens. *Front. Microbiol.* **7**, 1–18 (2016).
5. Rasmussen, R. Quantification on the LightCycler. in *Rapid Cycle Real-Time PCR* 21–34 (Springer Berlin Heidelberg, 2001). doi:10.1007/978-3-642-59524-0_3.
